# TPI1‐reduced extracellular vesicles mediated by Rab20 downregulation promotes aerobic glycolysis to drive hepatocarcinogenesis

**DOI:** 10.1002/jev2.12135

**Published:** 2021-08-11

**Authors:** Bonnie Hei Man Liu, Sze Keong Tey, Xiaowen Mao, Angel Po Yee Ma, Cherlie Lot Sum Yeung, Samuel Wan Ki Wong, Tung Him Ng, Yi Xu, Yue Yao, Eva Yi Man Fung, Kel Vin Tan, Pek‐Lan Khong, Daniel Wai‐Hung Ho, Irene Oi‐Lin Ng, Alexander Hin Ning Tang, Shao Hang Cai, Jing Ping Yun, Judy Wai Ping Yam

**Affiliations:** ^1^ Department of Pathology Li Ka Shing Faculty of Medicine The University of Hong Kong Hong Kong China; ^2^ Department of Hepatopancreatobiliary Surgery Second Affiliated Hospital of Harbin Medical University Harbin China; ^3^ Department of Endocrinology Second Affiliated Hospital of Harbin Medical University Harbin China; ^4^ Department of Chemistry State Key Laboratory of Synthetic Chemistry The University of Hong Kong Hong Kong China; ^5^ Department of Diagnostic Radiology Queen Mary Hospital the University of Hong Kong Hong Kong China; ^6^ State Key Laboratory of Liver Research (The University of Hong Kong) Hong Kong China; ^7^ Department of Infectious Diseases Nanfang Hospital Southern Medical University Guangzhou China; ^8^ Department of Pathology Sun Yat‐sen University Cancer Centre Guangzhou China

**Keywords:** extracellular vesicles, glycolysis, hepatocellular carcinoma, Rab20 GTPase, triosephosphate isomerase 1

## Abstract

Rab GTPases are major mediators that ensure the proper spatiotemporal regulation of intracellular trafficking. Functional impairment and altered expression of Rab proteins have been revealed in various human cancers. There is an emerging evidence about the role of Rab proteins in the biogenesis of extracellular vesicles (EVs). In hepatocellular carcinoma (HCC), using RNA sequencing comparing expression profiles of adjacent non‐tumorous tissues and HCC, Rab20 is identified to be the most frequently downregulated Rab member in HCC. Functionally, restoration of Rab20 in metastatic HCC cells results in the release of EVs with a diminished activity to promote cell growth, motility and metastasis. Conversely, EVs released from normal liver cells with Rab20 knockdown loses suppressive effect on HCC cell growth and motility. Proteomic profiling revealed the level of triosephosphate isomerase 1 (TPI1), a glycolytic enzyme, in EVs to be positively associated with Rab20 expression of the releasing cells. TPI1 targeted to be expressed in EVs released by Rab20 knockdown cells compromises the oncogenic activity of EVs. Besides, EVs released by TPI1 knockdown cells recapitulates the promoting effect of EVs derived from HCC cells with Rab20 underexpression. Aerobic glycolysis is beneficial to the survival and proliferation of tumour cells. Here, we observed that the enhanced cell growth and motility are driven by the enhanced aerobic glycolysis induced by EVs with reduced TPI1. The addition of glycolytic inhibitor blocks the promoting effect of EVs with reduced TPI1. Taken together, our study provides a mechanistic link among tumour cell‐derived EVs and glucose metabolism in HCC with Rab20 deregulation.

## 1 INTRODUCTION

Extracellular vesicle (EV) has become a new attraction in the field of cancer research by its emerging role in facilitating cancer progression. EVs are important nanosized communicators for both local and distant cell‐cell interaction through transferring various types of active biomolecules, such as proteins, lipids and RNA. These cargos are either evolutionary conversed or cell‐type specific (Meckes & Raab‐Traub, 2011). Cumulating evidence has delineated the prominent role of EVs in supporting cancer metastasis by means of modulating cell phenotypes and tumour microenvironment, in particular driving the formation of pre‐metastatic niche–the fertile ‘soil’ for seeding of cancer cells (Atay et al., 2014; Fong et al., 2015; Svensson et al., 2011). For example, highly metastatic pancreatic ductal adenocarcinoma cells release EVs with a high level of migration inhibitory factor that favours neoplastic transformation and epithelial‐mesenchymal‐transition modulation in naïve Kupffer cells to prime the liver for metastasis (Costa‐Silva et al., 2015). A comprehensive study has also uncovered the effect of tumour‐derived EV integrins on organotropic metastasis, and demonstrated lung‐ and liver‐tropic EVs that express differential integrin expression patterns to direct organ‐specific metastasis (Hoshino et al., 2015). These promising studies offer insight into the importance of an in‐depth understanding of the engagement of EVs in carcinogenesis.

Extracellular vesicle synthesis relies on several families of proteins, including the Rab GTPases (Ostrowski et al., 2010). With over 70 members, Rab GTPases are well‐studied key mediators of membrane trafficking, controlling vesicle cargo sorting, uncoating, motility, tethering and membrane fusion (Stenmark, 2009). Activation of Rab proteins relies on the binding of nucleotide guanosine triphosphate and subsequent conformational change at the switch regions, leading to their interplay with the effector molecules for functioning in a precise manner (Delprato et al., 2004; Eathiraj et al., 2005). Besides their roles in intracellular trafficking, involvement of Rab proteins in carcinogenesis are also explored. Aberrant expression of Rab proteins modifies cancer development and metastasis, in terms of tumour cell proliferation, invasiveness and resistance to drug therapy (Recchi & Seabra, 2012).

In this study, we focused on exploring the functions of Rab proteins in hepatocellular carcinoma (HCC) pathogenesis. HCC, the dominant type of liver cancer, is characterized by its complicated reprogramming in both genetic and cytological features in the cancerous hepatocytes (Farazi & Depinho, 2006). Despite the emergence of several tyrosine kinase and immune checkpoint inhibitors for treating HCC, it still remains as one of the deadliest cancers worldwide due to delayed diagnosis of the disease (Bray et al., 2018). Although metastasis has been recognized as a hallmark of cancer for over a decade, its molecular mechanism remains poorly understood due to the complex involvement of biomolecules and signalling pathways involved with the process (Hanahan & Weinberg, 2011). Therefore, uncovering the dynamic mode of metastasis in HCC can provide a new perspective on understanding the complexity of hepatocarcinogenesis, and hopefully aid the identification of new therapeutic targets for HCC patients.

Here, we identified that Rab20 is frequently underexpressed in HCC. Rab20 is a 26 kDa small GTPase, sharing similar sequence identity with endocytic Rab proteins (Stein et al., 2012). Studies on Rab20 mainly concentrated on delineating its role in immune regulation (Gutierrez et al., 2008; Liang et al., 2012; Torri et al., 2010). It is involved in controlling endosome maturation in macrophages (Pei et al., 2015), as well as phagosome biogenesis during bacterial infection (Egami & Araki, 2012; Schnettger et al., 2017). However, its role in cancer remains obscure. We found that rescuing Rab20 in HCC cells abrogated the ability of cell growth, cell motility and tumour formation. Apart from enhancing cell aggressiveness, Rab20 underexpression resulted in the secretion of EVs with an enhanced capacity in promoting cell aggressiveness *in vitro* and metastasis in animals. Proteomic profiling identified the reduced level of triosephosphate isomerase 1 (TPI1) in EVs released by HCC cells with Rab20 underexpression. Restored TPI1 level in EVs inhibited the promoting activity of EVs. Mechanistically, the findings showed that TPI1‐reduced EVs enhanced aerobic glycolysis leading to the enhanced growth and motility of the recipient cells. This study has uncovered an unrecognized function of Rab20 and its association with EV activities in HCC carcinogenesis, and revealed the functions of HCC cell‐derived EVs in the induction of aerobic glycolysis in tumour cells.

## 2 MATERIALS AND METHODS

### 2.1 Clinical HCC samples

Fifty pairs of tumorous and adjacent non‐tumorous liver tissue were collected from patients who have undergone surgical operations at Queen Mary Hospital, Hong Kong. RNA was isolated from the specimen for mRNA expression analysis. Procedures involving human specimen were conducted with the approval of the Institutional Review Board of The University of Hong Kong/ Hospital Authority Hong Kong West Cluster (HKU/HA HKW IRB). A total of 141 pairs of HCC specimens and adjacent liver tissues were obtained from tissue blocks filed in the Department of Pathology, Sun Yat‐sen University Cancer Centre. Each tissue block was sliced (diameter: 0.6 mm) from the marked area and re‐embedded using a tissue array instrument (Minicore Excilone, Minicore). Fixation of specimens was done in 4% paraformaldehyde/0.1 M phosphate buffer followed by embedment in paraffin wax. Embedded tissues were sliced into 9 μm sections and mounted onto glass slices.

### 2.2 Cell lines and cell culture

Human embryonal kidney cell line HEK293FT, human umbilical vein endothelial cells (HUVEC), immortalized liver cell line MIHA, and HCC cell lines Hep3B, HLE, Huh7 and PLC/PRF/5 were purchased from American Type Culture Collection. Metastatic HCC cell line MHCC97L and MHCCLM3 were given by Fudan University. p53^–/–^;Myc‐transduced murine hepatoblasts was a gift from Scott Lowe, Memorial Sloan Kettering Cancer Centre, New York (Xue et al., 2012). Except HUVEC, all cell lines were cultured with 10% fetal bovine serum (FBS)‐supplemented DMEM‐HG medium supplemented with 100 U/ml penicillin‐streptomycin. HUVEC was cultured in LSGS‐supplemented Medium 200 with 100 U/ml penicillin‐streptomycin.

To establish stable knockdown and overexpression of Rab20 and TPI1 in MIHA and luciferase‐labelled MHCC97L cell lines, respectively, either FuGene 6 transfection reagent (Promega) or EndoFectin‐Lenti (GeneCopeia) was used for generating viral particles in HEK293FT cells. Rab20 overexpression plasmid was purchased from GeneCopeia, while short‐hairpin RNAs (shRNAs) for both Rab20 and TPI1 were purchased from Sigma‐Aldrich. To construct plasmid for overexpressing TPI1 in EVs, full length TPI1 was amplified from Human TPI1 Gene ORF CDNA clone (Sino Biological) by PCR using forward primer: 5′‐CGGGATCCACATGGCGCCCTCCAGGAA‐3′ and reverse primer: 5′‐AACTGCAGTCATTGTTTGGCATTGATGATG‐3′. The amplified TPI1 PCR fragment was inserted into XPack CMV‐XP‐MCS‐EF1‐Puro cloning lentivector (System Biosciences). The XPack‐TPI1 vector was confirmed by DNA sequencing. Viral particles were collected from XPack‐TPI1‐transfected HEK293FT cells and used to infect target cell lines. Infected cells were selected with puromycin at the concentration of 1 μg/ml.

### 2.3 Isolation and validation of EVs

Cell culture seeded at a density of 5 × 10^7^ cells per 150‐mm plate was incubated with medium supplemented with 10% EV‐free FBS for 72 h. EV‐free FBS was prepared by centrifuging FBS overnight at 100,000 × g at 4°C (Beckman Coulter, Avanti JXN‐30). EVs were isolated from the 300 ml conditioned medium (CM) by sequential ultracentrifugation. CM was collected and centrifuged at 3000 × g for 15 min, followed by a subsequent centrifugation at 20,000 × g for 30 min. The supernatant was filtered through a 0.22 μm syringe filter (Millipore), and centrifuged at 100,000 × g for 2 h using JA‐30.50Ti fixed angle rotor (Beckman Coulter). After centrifugation, the supernatant obtained was regarded as ‘EV‐free’ CM. The pellet was washed with PBS and centrifuged at 100,000 × g for 75 min. The isolated EV pellet was resuspended in PBS, and stored at ‐20°C until use.

The EV pellet was resuspended in PBS and subjected to nanoparticle tracking analysis by nanoparticle tracking video microscope ZetaView TWIN – NTA Nanoparticle Tracking–Video Microscope PMX‐220 (Particle Metrix GmbH). Videos at 11 distinct locations in the cell assembly were recorded. Data analysis was performed with software ZetaView (version 8.05.11). EV pellet was lysed using NETN lysis buffer (0.1% NP40, 25 mM Tris‐HCl, 50 mM NaCl2, 0.2 mM EDTA). The protein lysates of EVs were subjected to western blot analysis using antibodies against Alix (Santa Cruz Biotech), TSG101 (BD Biosciences), CD9 (Abcam), GM130 (Abcam) and nucleoporin p62 (Cell Signalling Technology). Procedure of immunogold labelling of EVs was modified from the protocol described by Thery et al (Théry et al., 2006). In brief, 10 μg of EVs was deposited on an activated Formvar‐carbon coated electron‐microscopy grid. The grid was washed with PBS, incubated with PBS/50 mM glycine and blocked with 1% BSA in PBS. The grid was then incubated with anti‐CD63 antibody (Abcam), washed and incubated with goat anti‐rabbit IgG H&L (5 nm gold) pre‐adsorbed (Abcam). The immunoreaction was stabilized by blocking the grid with 1% glutaraldehyde, washed and stained with 2% aqueous uranyl acetate, followed by counterstaining with Reynold's lead citrate. After drying, the grid was observed under Philips CM100 Transmission Electron Microscope attached with an Olympus SIS Tengra CCD Camera. We have submitted all relevant data of our experiments to the EV‐TRACK knowledgebase (EV‐TRACK ID: EV200160) (Consortium et al., 2017).

### 2.4 Subcutaneous and tail vein injection in mouse

Both subcutaneous and tail vein injection were performed in male BALB/C nude mice. HCC cells of 1 × 10^6^ suspended in PBS were subcutaneously injected into nude mice. Tumour size was monitored and measured weekly for 4 weeks. Volume of tumour was calculated as follows: 0.5 × (L × W^2^), where L and W was the length and width of the tumour, respectively. EVs used for tail‐vein injection were isolated based on the procedures mentioned in the section Isolation and validation of EVs. Isolated EVs were diluted into 1 μg/μl with PBS and stored at ‐20°C until the day of injection. Suspensions of 1 × 10^5^ p53^–/–^;Myc‐transduced murine hepatoblasts together with or without 10 μg EVs were injected into each nude mouse via intravenous tail‐vein injection. Mice were sacrificed 2 weeks after the injection. Bioluminescence signals emitted by the lungs before and after excision were visualized by IVIS Spectrum In Vivo Imaging System (PerkinElmer).

### 2.5 Orthotopic implantation in liver and micro‐PET scan

EV‐treated Huh7 cells of 1 × 10^6^ were suspended in 15 μl Matrigel, and injected into the left lobe of the liver in each 6‐week‐old nude mouse. Four weeks post injection, mice were subjected to ^18^F‐FDG PET scan on the pre‐clinical nanoScan PET/MRI system (Mediso Medical Imaging Systems Ltd). One day prior to the scan, mice were fasted overnight with free access to water *ad libitum*. Mice were maintained under anaesthesia by inhaling 5% isoflurane/oxygen mixture and placed in the prone position on the pre‐heated animal bedding of the scanner equipped with heating system to monitor and maintain body temperature in between 36.5 and 37.5°C throughout the imaging process. Respiratory rate was also monitored and recorded by the integrated respiratory monitoring system of the scanner. The tail of each mouse was warmed gently using warm water bath immediately before injection of ^18^F‐FDG (7.28 ± 0.26 MBq) via intravenous tail‐vein injection. Mice were maintained using 2% isoflurane/oxygen during the 60 min uptake and imaging acquisition periods to reduce the non‐specific background uptake. A whole body, static PET acquisitions were performed for 20 min in 1:3 coincidence and normal count mode. T1‐ and T2‐weighted MRI scans were performed for anatomical information and attenuation correction of PET images. The PET images were reconstructed using Tera‐Tomo 3D (OSEM), with radionuclide decay, normalization, random, scatter, attenuation and dead time corrections applied to the data, resulting in a matrix of 0.3 mm^3^. Both PET and MR images were co‐registered automatically, and data analyses were performed using InterView Fusion (Mediso Ldt., Budapest, Hungary). For each scan, the volume of interest (VOI) of tumour or liver (with a radius of 3 mm) were drawn manually in the PET images and presented as the standard uptake value (SUV). SUV was calculated using the equation: SUV = C_PET_(*t*)/(ID/BW), where C_PET_(*t*) is the measured activity in VOI, ID is the injected dose measured in kBq, and BW is the mouse body weight in kg, assuming a tissue density of 1 g/ml.

### 2.6 Liquid chromatography tandem MS (LC‐MS/MS) and data analysis

Isolated EVs were lysed in 8 M urea/100 mM Tris‐HCl buffer, and incubated at 60°C for 10 min. A final concentration of 5 mM dithiothreitol was added to the samples and incubated for 20 min at room temperature. A final concentration of 25 mM iodoacetamide was subsequently added and the samples were incubated in the dark for 30 min. Trypsin in 1 M urea was added to the samples at a ratio of 1:50 (trypsin:protein) and incubated at 37°C for 16h. The proteolysis was quenched by adding 5% formic acid. Digested samples were desalted by C18 STAGE tips and concentrated using SpeedVac (Thermo Savant).

Digested protein samples were analysed with a UPLC‐MS/MS setup. The analytic column was a 25 cm column (360 μm outer diameter, 50 μm inner diameter, 1.9 μm C18 packing material, Pepsep). The mobile phases composed of A (0.1% formic acid in water) and B (0.1% formic acid in 80% ACN). Each sample carrying 2 μg peptides was loaded onto the analytical column by the auto‐sampler of the RPLC (EASY‐nLC 1200, Thermo Scientific). The column was eluted with a gradient of 5% to 10% B for 10 min, followed by a gradient of 10–15% B for 60 min, and finally a gradient of 15–28% B for 63 min at a flow rate of 150 nl/min. For the MS analysis, Orbitrap Fusion Tribrid Mass Spectrometer (Thermo Scientific) was operated in a data‐dependent mode cycling through a high‐resolution (120,000 at 400 *m/z*) full scan MS^1^ (350–1,500 *m/z*) followed by HCD MS^2^ scan on the most abundant ions from the immediately preceding full scan in a cycle time of 3 s. The selected ions were isolated with a 1.6‐Da mass window and put into an exclusion list for 60 s after they were first selected for HCD.

Using MaxQuant search engine (version 1.6.5.0), raw files generated during LC‐MS/MS analysis were searched against the Uniprot Human database (downloaded on 5 Nov 2019, 178,281 entries). The search was specified to trypsin digestion (allowed up to two missed cleavages), oxidation of methionine as a dynamic modification, and iodoacetamide derivative of cysteine as a static modification. The mass tolerance for MS^1^ for first and main search was 20 ppm and 4.5 ppm, respectively, while it was 20 ppm for MS^2^. With a decoy search strategy, the peptide false discovery rate (FDR) was set to 1%. EV proteins of Rab20 cells with fold change ≥ 2‐fold and ≤ 0.5 when compared to Vector cells was regarded as upregulated or downregulated, respectively.

### 2.7 Statistical analysis

Statistical analysis for both *in vitro* and *in vivo* assays were performed with GraphPad Prism 8 (USA). *P*‐value was calculated by unpaired student *t*‐test for most of the *in vitro* and *in vivo* assays. For *in vitro* assays determining the effect of reduction in TPI1 level in shRab20‐EV, ANOVA was applied to calculate the *P*‐value. *P* < 0.05 was regarded as statistically significant.

## 3 RESULTS

### 3.1 Rab20 is frequently downregulated in HCC

**FIGURE 1:**
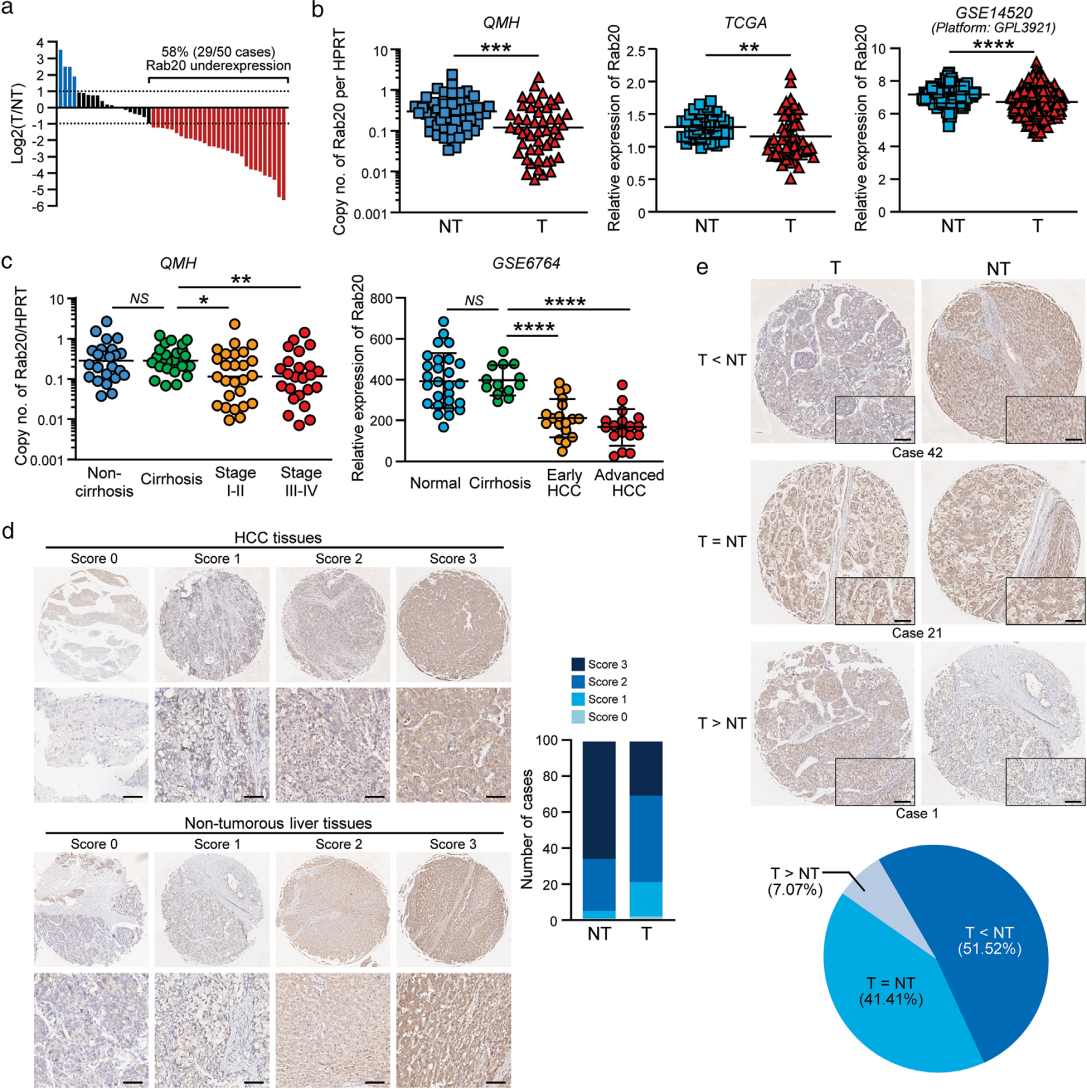
Rab20 is frequently downregulated in HCC clinical samples. (a) The waterfall plot shows the fold change of Rab20 expressions in 50 pairs of HCC and adjacent non‐tumorous tissues in log scale. (b) Expressions of Rab20 in HCC and non‐tumorous liver tissue were compared in cohorts from Queen Mary Hospital (QMH) (*left*), TCGA (*middle*) and GSE14520 (platform: GPL3921) (*right*) databases of liver cancer. (c) Dot plots showing expression of Rab20 in samples from QMH (*left*) and GSE6764 (*right*) database in a stage‐dependent manner during HCC development. (d) Immunohistochemical staining of Rab20 in TMA. Representative images of Rab20 expression in HCC and non‐tumorous liver tissues with high (scores 2 to 3) and low (0 to 1) scores. Scale bar: 50 μm. The bar chart showing number of cases based on scoring intensity. A total of 99 cases of paired HCC and non‐tumorous tissues were stained. (e) Representative cases showing overexpression (case 42), no change (case 21) and underexpression (case 1) of Rab20. The pie chart reveals Rab20 underexpression in about half of the cases. Data are represented as mean. *P < 0.05, **P < 0.01, ***P < 0.001, ****P < 0.0001. *P* < 0.05 is considered as statistically significant. *NS*, not significant

Nine members of the Rab GTPase family were examined for their expressions in a paired sample of HCC tissues and non‐tumorous liver tissue (**Figure S1A**). Four of the deregulated Rab proteins, Rab3D, Rab20, Rab30 and Rab42, were further tested for their expressions in 10 clinical cases using quantitative RT‐PCR (**Figure S1B**), among which, Rab20 was validated to be significantly downregulated. The study cohort was further expanded to 50 cases collected from HCC patients hospitalized in Queen Mary Hospital (QHM). Rab20 was downregulated by at least two folds in 58% (29/50) of the cases (Figure 1a). Rab20 downregulation was also shown in databases of liver cancer extracted from The Cancer Genome Atlas (TCGA) and Gene Expression Omnibus (GEO) platforms, suggesting that Rab20 downregulation was a common event in HCC pathogenesis (Figure 1b). As revealed in the cohort of HCC cases of QMH and GSE database, Rab20 downregulation in HCC occurred in the early stage of HCC (Figure 1c). We also performed tissue microarray to examine protein expression of Rab20 in HCC. Out of 99 cases, score 3 was detected in 65 non‐tumorous tissues compared to 30 HCC tissues (Figure 1d). Rab20 downregulation was found in 51.52% (51/99 cases) of cases examined (Figure 1 **E)**; its downregulation was also observed in TCGA and Genotype‐Tissue Expression (GTEx) portal via Gene Expression Profiling Interactive Analysis (GEPIA) platform of other solid tumours (Figure S2).

### 3.2 Rab20 confers the suppressing role in HCC cell growth, motility and tumorigenesis

**FIGURE 2:**
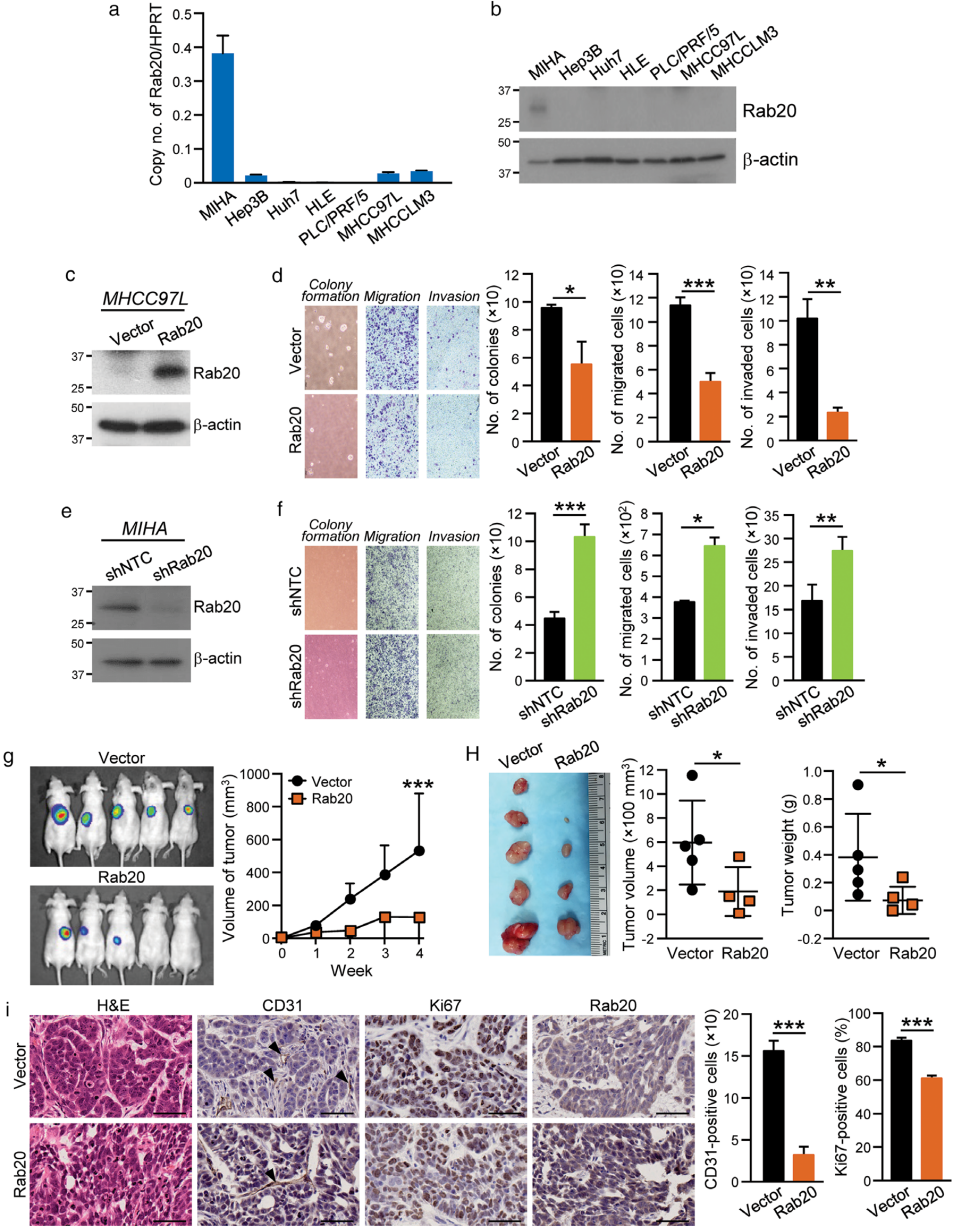
Rab20 negatively regulates growth, motility and tumorigenic potentials of HCC cells. (a‐b) mRNA and protein levels of Rab20 in MIHA and HCC cell lines were quantified by quantitative RT‐PCR and immunoblotting, respectively. (c) MHCC97L was stably transduced with Rab20 expression plasmid and empty vector, and immunoblotted with anti‐Rab20 and anti‐β‐actin antibodies. (d) MHCC97L Vector and Rab20 cells were subjected to soft agar (*Left*), migration (*Middle*) and invasion (*Right*) assays. Number of colonies, migrated and invaded cells were counted. Representative images of colonies and cells are shown. (e) Immunoblot image showing the expression of Rab20 in MIHA stably transduced with non‐targeted shRNA control (shNTC) and shRNA targeting Rab20 (shRab20). (f) MIHA shNTC and shRab20 cells were subjected to soft agar (*Left*), migration (*Middle*) and invasion (*Right*) assays. Number of colonies, migrated and invaded cells were counted. Representative images of colonies and cells are shown. (g) MHCC97L Vector and Rab20 cells were subcutaneously injected into nude mice. Tumour growth rate was monitored and measured weekly. Four weeks after injection, bioluminescence signals emitted by the subcutaneous tumours were measured. (h) Volume and size of the excised xenograft were recorded and compared. (i) Paraffin‐embedded tissues obtained from xenograft were stained with H&E, anti‐CD31, anti‐Ki67 and anti‐Rab20 antibodies. Arrow indicates the presence of stained blood vessels. The numbers of CD31‐ and Ki67‐positive cells were quantified and plotted. Scale bar: 100 μm. Data are represented as mean ± SEM. *P < 0.05, **P < 0.01, ***P < 0.001. *P* < 0.05 is considered as statistically significant

In accordance with the expression of Rab20 in HCC tissues, Rab20 was highly expressed in immortalized hepatocyte cell line MIHA while it was barely detected in non‐metastatic HCC cell lines (Hep3B, Huh7, HLE and PLC/PRF/5) and metastatic HCC cell lines (MHCC97L and MHCCLM3) (Figure 2a and 2b). To investigate the functions of Rab20 in HCC, Rab20 was stably expressed in metastatic luciferase‐labelled MHCC97L cells (Figure 2c). Overexpressing Rab20 significantly reduced the number of colonies formed, implying the suppression of anchorage‐independent growth by Rab20. Rab20 overexpression also significantly inhibited cell migration and invasion (Figure 2d). Mirroring the effect of overexpressing Rab20, silencing of Rab20 was performed in normal liver cell line MIHA (Figure 2e). Rab20 knockdown promoted the aggressiveness of cells by promoting anchorage‐independent growth, migratory and invasive abilities (Figure 2f), which phenocopied HCC development in normal liver cells. *In vivo* effect of overexpressing Rab20 in HCC tumorigenesis was examined by subcutaneous injection of control Vector and Rab20 cells in immune‐deficient mice. Rab20 overexpressing cells suppressed tumour development with smaller tumours formed when compared to Vector cells (Figure 2g and 2h). IHC revealed strong Rab20 expression in xenografts derived from Rab20 cells. Tumours derived from Rab20 cells showed reduced expressions in the angiogenic marker CD31 and Ki67, suggesting reduced angiogenic and proliferative abilities of the tumour cells (Figure 2i). These findings indicated that the functions of Rab20 is congruent with the clinical data of Rab20 expression in HCC.

### 3.3 Intracellular Rab20 expression modulates activities of the releasing EVs

**FIGURE 3:**
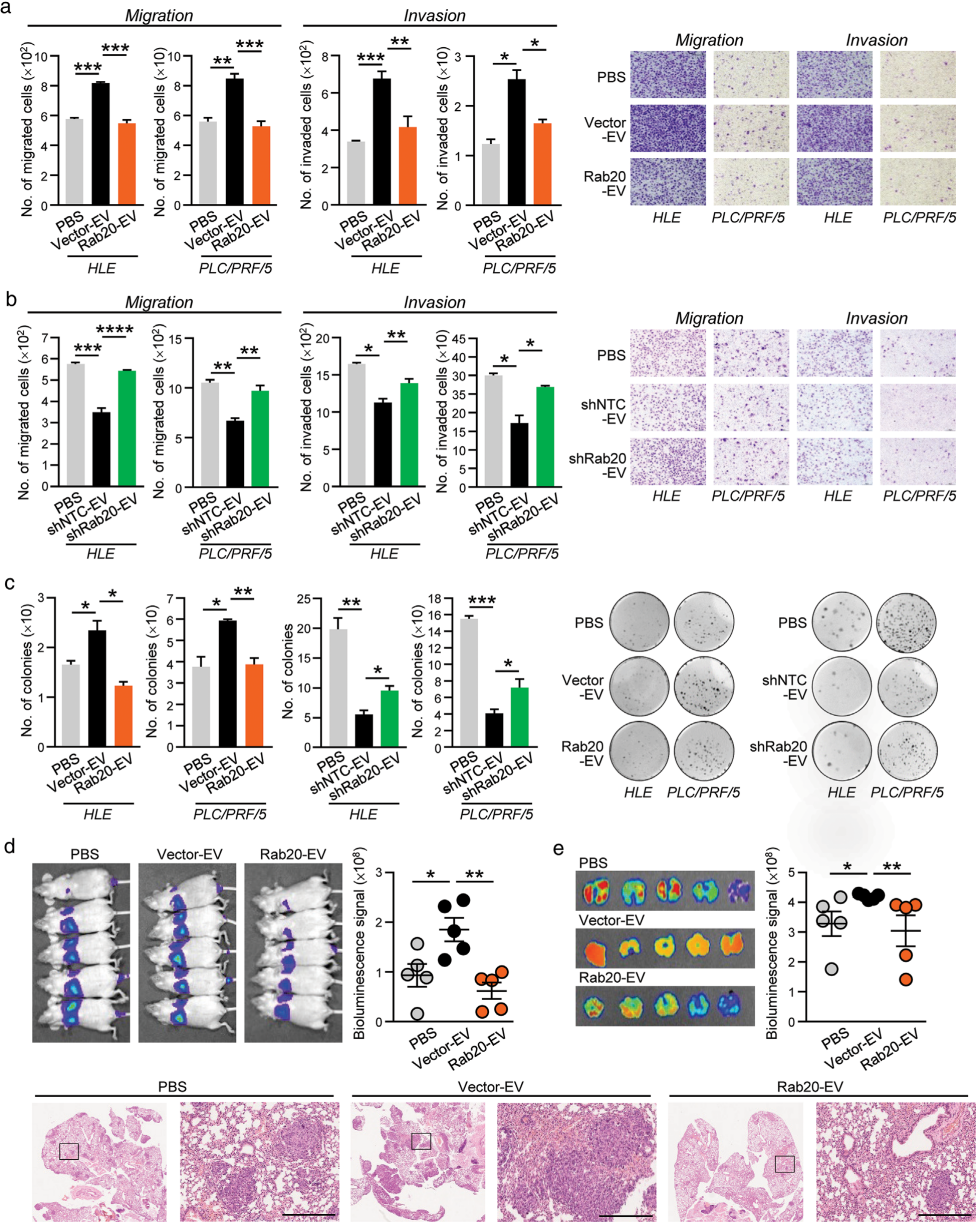
Diminished promoting activity of EVs released by MHCC97L cells with Rab20 overexpression. HLE and PLC/PRF/5 cells were treated with MHCC97L Vector‐ and Rab20‐EV (a), and MIHA shNTC‐ and shRab20‐EV (b). Treated HCC cells were seeded for migration and invasion assays. Representative images were shown and the number of cells was quantified. (c) Colony formation assay was performed on HLE and PLC/PRF/5 cells treated with the indicated EVs. Colonies formed were fixed, stained and counted. (d) p53‐/‐;Myc‐transduced mouse hepatoblasts were co‐injected with PBS, MHCC97L Vector‐ and Rab20‐EV in nude mice through tail vein. Two weeks after co‐injection, bioluminescence imaging of animals was performed. (e) *Ex vivo* bioluminescence imaging of the excised lungs was performed. Representative H&E‐stained images showing tumour nodules in the lungs were shown. Scale bar: 500 μm. Data are represented as mean ± SEM. *P < 0.05, **P < 0.01, ***P < 0.001, ****P < 0.0001. *P* < 0.05 is considered as statistically significant

Owing to the role of Rab20 in controlling membrane trafficking and vesicle cargo sorting, we speculated that Rab20 is involved with the regulation of the secretory pathway or EV production in HCC. Conditioned medium (CM), EV‐free CM and isolated EVs were collected from MHCC97L Vector and Rab20 cells. The isolated EVs were validated for their identity and purity. EVs were shown to express the positive EV markers Alix and TSG101, but not the negative markers GM130 (Golgi marker) and p62 (nuclear marker) (**Figure S3A**). Alix and TSG101 are involved in cargo sorting during the early step of EV biogenesis by acting as the interacting partners of the endosomal sorting complex required for transport (ESCRT) complexes (Raiborg & Stenmark, 2009). Immunogold‐labelling TEM showed that EVs were positively stained with CD63, an EV marker (**Figure S3B**). The size range of EVs was within the reference range of exosomes (**Figure S3C**). We adopted the guidelines of MISEV2018 (Théry et al., 2018), and used the generic term EV in our study. HLE and PLC/PRF/5 cells treated with CM, EV‐free CM and isolated EVs were compared for their migratory and invasive abilities. CM and EVs of Rab20 cells (Rab20‐EV) exerted suppressive effect in cell motility when compared to CM and EVs of Vector cells (Vector‐EV), respectively. Depletion of EV from CM posed no effect in cell motility, which substantiated that the suppressive effect was dependent on EVs (Figure 3a; Figure S4A and S4B). The suppressive effect was also observed in the colony formation ability of HCC cells (Figure 3c), reflecting that Rab20 expression in metastatic MHCC97L cells resulted in the release of less aggressive EVs. Similarly, CM, EV‐free CM and isolated EVs were collected from MIHA non‐target control (shNTC) and Rab20 knockdown (shRab20) cells and subjected to functional characterization. CM and EVs collected from shRab20 cells (shRab20‐EV) significantly enhanced the motility of HLE and PLC/PRF/5 cells, while EV‐free CM posed no effect when compared to EV of shNTC cells (shCTL‐EV) (Figure 3b; Figure S4C and S4D). shNTC‐EV inhibited colony formation of HCC cells; such inhibitory effect was partially lost in shRab20‐EV (Figure 3c). In an experimental metastasis assay, MHCC97L Vector‐EV significantly facilitated the colonization of p53^–/–^;Myc‐transduced murine hepatoblasts in lungs upon intravenous co‐injection. However, such enhancement was not seen in mice injected with hepatoblasts with Rab20‐EV (Figure 3d and 3e). As tumours derived from Rab20 overexpressing cells showed reduced angiogenic potential (Figure 2i), the ability of EV released by cells with Rab20 deregulation in angiogenesis was explored. HUVEC was shown to be able to uptake EVs derived from tumour cells after incubation (**Figure S5A**). EVs released by MIHA with Rab20 knockdown were shown to enhance tube formation ability and sprouting of HUVEC. Such promoting effect of EVs was lost in EVs obtained from control cells (**Figure S5B and S5C**). Consistent effect of EVs obtained from Rab20 knockdown cells in enhancing the formation of microvessels in tumours was seen in matrigel plug *in vivo* angiogenesis assay (**Figure S5D** **)**. Taken altogether, these results implied that the activity of EVs was a functional mediator of Rab20 underexpression‐driven tumorigenesis.

### 3.4 Identification of TPI1 as a functional component in EVs regulated by Rab20 expression

**FIGURE 4:**
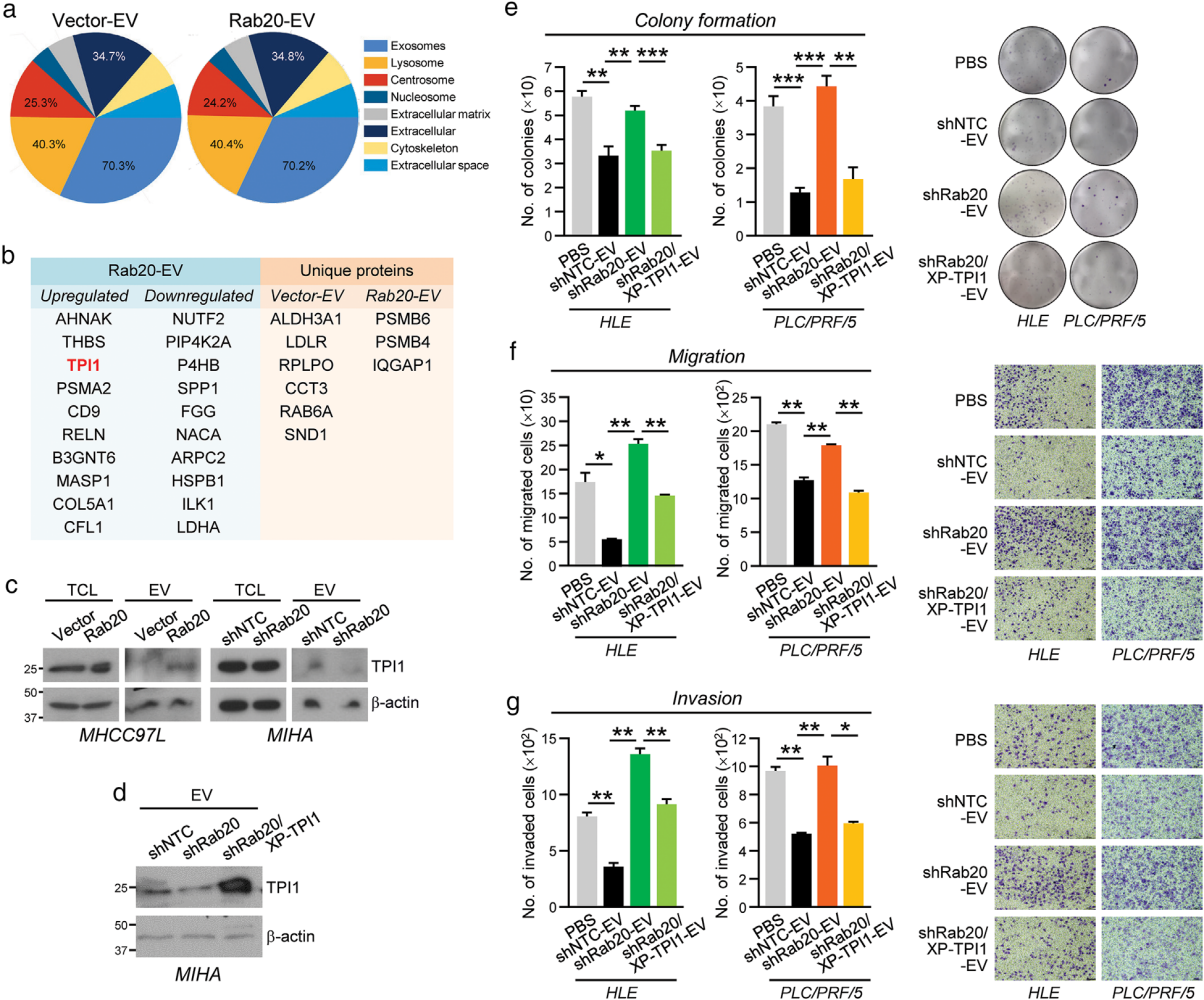
Identification of TPI1 in EV regulated by intracellular Rab20 expression. (a) Proteins isolated from EVs derived from MHCC97L Vector‐ and Rab20‐EV were analysed by mass spectrometry. Cellular distribution of the identified proteins was analysed by FunRich 3.1.3, and displayed in the pie chart. (b) Rab20‐EV proteins with a fold change of at least 2‐fold when compared to Vector‐EV was regarded as upregulated or downregulated. The top 10 significantly modulated proteins in Rab20‐EV when compared to Vector‐EV (*Left*), and uniquely expressed proteins in Vector‐ and Rab20‐EV (*Right*) are shown. (c) Expression of TPI1 was probed with anti‐TPI1 antibody in the total cell lysates (TCL) and EVs of MHCC97L Vector and Rab20 cells (*left*), as well as MIHA shNTC and shRab20 cells (*right*). β‐actin was used to normalize the loading of protein samples. (d) Immunoblot image showing the expression of TPI1 in MIHA shCTL‐, shRab20‐, and shRab20/XPack‐TPI1‐EV. HLE and PLC/PRF/5 cells treated with indicated EVs were subjected to colony formation (e), migration (f) and invasion (g) assays. At the end of incubation, colonies, migrated and invaded cells were fixed, stained and counted. Representative images of cells and colonies were displayed. Data are represented as mean ± SEM. *P < 0.05, **P < 0.01, ***P < 0.001. *P* < 0.05 is considered as statistically significant

Subsequent to the functional characterization of EVs from releasing cells modulated by Rab20 expression, mass spectrometry analysis was applied to determine the proteomic profiles of MHCC97L Vector‐ and Rab20‐EV (**Table S1**). Approximately 70% of EV proteins comprised exosome constituents, which correlated with the nature of the sample (Figure 4a). Differential expression profile of proteins was detected in Vector‐ and Rab20‐EV (Figure 4a), in which 20 proteins were upregulated while 13 proteins were downregulated by at least 2‐fold and with *P*‐value less than 0.05 (**Table S2**). The top 10 upregulated and downregulated proteins in Rab20‐EV, as well as proteins uniquely expressed in either Vector‐ or Rab20‐EV, were listed in Figure 4b. The top listed candidates were examined for their expressions in Vector‐ and Rab20‐EV. Among the validated candidates, only Triosephosphate isomerase 1 (TPI1), the third upregulated protein in Rab20‐EV, was found to be reduced in EVs derived from MIHA shRab20 cells (Figure 4c). These results indicated that EV‐TPI expression was positively correlated with the intracellular Rab20 expression. Intriguingly, the level of cellular TPI1 expression remains unchanged in Rab20 overexpressing and knockdown cells. TPI1 is a homodimer glycolytic enzyme that catalyses the isomerization of glyceraldehyde‐3‐phosphate (G3P) and dihydroxyacetone phosphate (DHAP) in glycolysis. Despite the fact that TPI1 seems to have a contradictory function in cancer (Unwin et al., 2003; Wang et al., 2008; Zhang et al., 2009), its inhibitory role in HCC has been reported (Jiang et al., 2017).

### 3.5 Restoration of TPI1 reduces the promoting effect of EVs released by Rab20 knockdown cells

To explore whether the functions of EVs mediated by Rab20 is dependent on TPI1, TPI1 was expressed and packaged into MIHA shRab20‐EV by utilizing an expression vector carrying an EV‐targeting peptide (XP). Immunoblotting confirmed the rescued TPI1 expression in EV of shRab20/XP‐TPI1 cells (shRab20/XP‐TPI1‐EV) (Figure 4d). The integrity and size of shNTC‐, shRab20‐ and shRab20/XP‐TPI1‐EV were validated prior to their examination using functional assays (Figure S6). Consistently, compared with PBS‐treated HLE and PLC/PRF/5 cells, cells treated with shNTC‐EV showed reduced abilities in colony formation, migration and invasion (Figure 4e‐4g). The shRab20‐EV with reduced TPI1 expression lost the inhibitory effect. Restored TPI1 expression in shRab20‐EV partially rescued the inhibitory activity of EVs on cell growth and motility.

**FIGURE 5:**
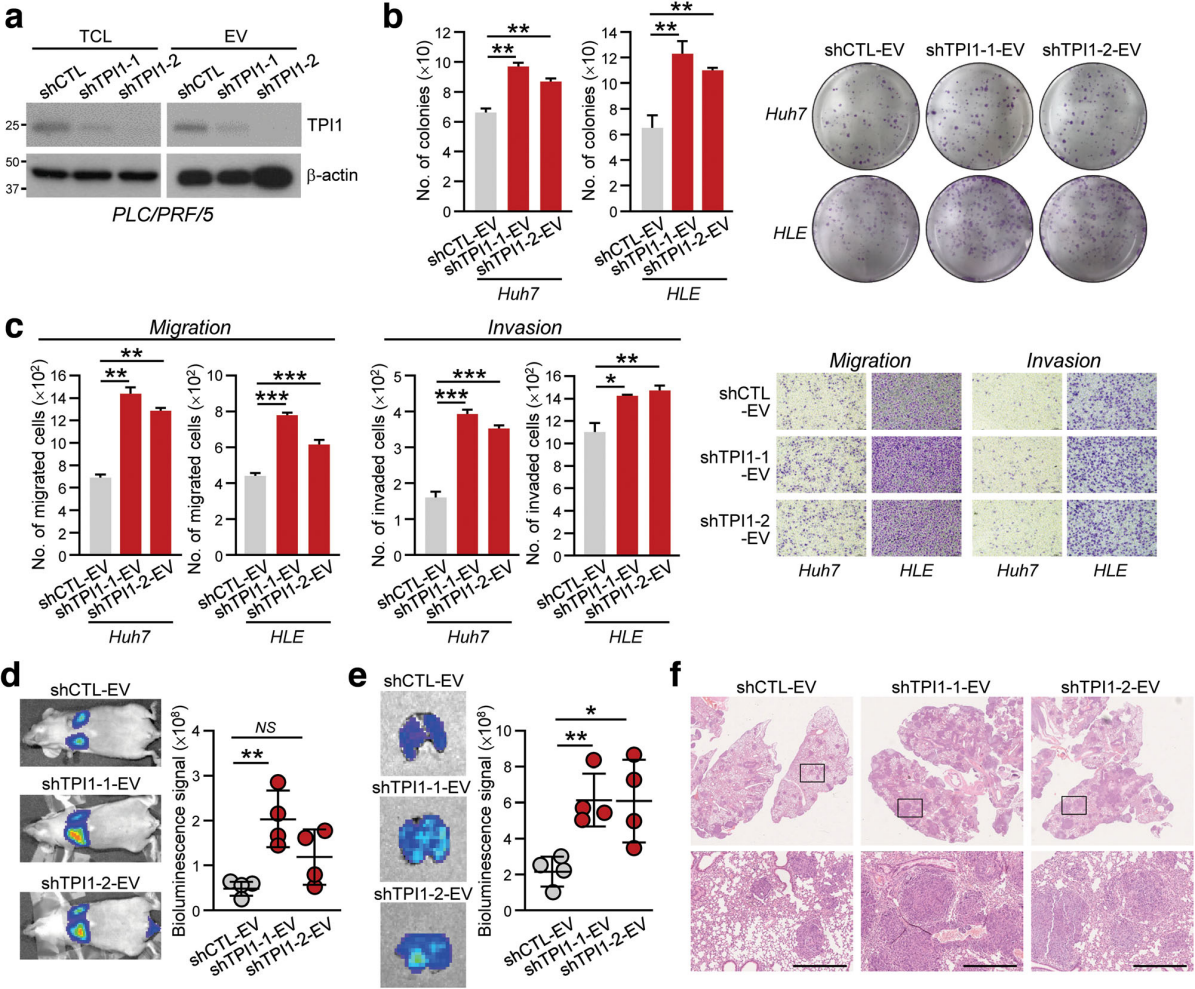
EVs with reduced TPI1 expression promoted aggressiveness of HCC cells. (a) PLC/PRF/5 cells were stably transduced with control shRNA (shCTL) and shRNA targeting TPI1 (shTPI1‐1 and shTPI1‐2). Total cell lysates (TCL) and isolated EVs were immunoblotted with anti‐TPI1 and anti‐β‐actin antibodies. (b) Huh7 and HLE treated with shCTL‐, shTPI1‐1 and shTPI1‐2‐EVs were subjected to colony formation assay. (c) Migratory and invasive abilities of Huh7 and HLE cells treated with shCTL‐, shTPI1‐1 and shTPI1‐2‐EVs were determined by migration and invasion assays. (d) Two weeks after co‐injection of p53‐/‐;Myc‐transduced murine hepatoblasts with shCTL‐, shTPI1‐1 and shTPI1‐2‐EVs, mice were subjected to bioluminescence imaging. Representative images of animals of each group are shown. (e) Lungs were excised and subjected to bioluminescence signal detection. (f) Representative H&E‐stained images showing tumour nodules in the lungs were shown. Scale bar: 500 μm. Data are represented as mean ± SEM. *P < 0.05, **P < 0.01, ***P < 0.001. *P* < 0.05 is considered as statistically significant

To affirm the functional role of EV‐TPI1, knockdown of TPI1 was conducted in PLC/PRF/5 cells to generate EVs with reduced TPI1 expression (shTPI1‐1‐EV and shTPI1‐2‐EV) (Figure 5a). EVs of non‐target control (shCTL‐EV) and TPI1 knockdown cells were validated for their identity, morphology and size (**Figure S7**). shTPI1‐EV markedly enhanced the abilities of HLE and Huh7 cells to form colonies, migrate and invade when compared to shCTL‐EV‐treated cells (Figure 5b and 5c). *In vivo* colonization ability of p53^–/–^;Myc‐transduced murine hepatoblasts was also evoked by co‐injection of hepatoblasts and shTPI1‐EVs (Figure 5d‐5f). These data showed that EV with reduced TPI1 recapitulated the functions of EVs released by cells with Rab20 knockdown. Conversely, XP‐TPI1 was overexpressed in MHCC97L cells to obtain TPI1‐enriched EV released by these cells (**Figure S8**). Resembling the enhanced growth and motility of cells induced by MHCC97L Vector‐EV, control XP‐CTL‐EV significantly enhanced the migratory, invasive and proliferative abilities of Huh7 and PLC/PRF/5 cells and the colonization of murine fibroblasts in the lungs (**Figure S9**). However, such enhancement was abolished when cells were treated with TPI‐enriched EVs of MHCC97L cell.

### 3.6 EVs with reduced TPI1 promoted aerobic glycolysis leading to enhanced growth and motility in recipient cells

**FIGURE 6:**
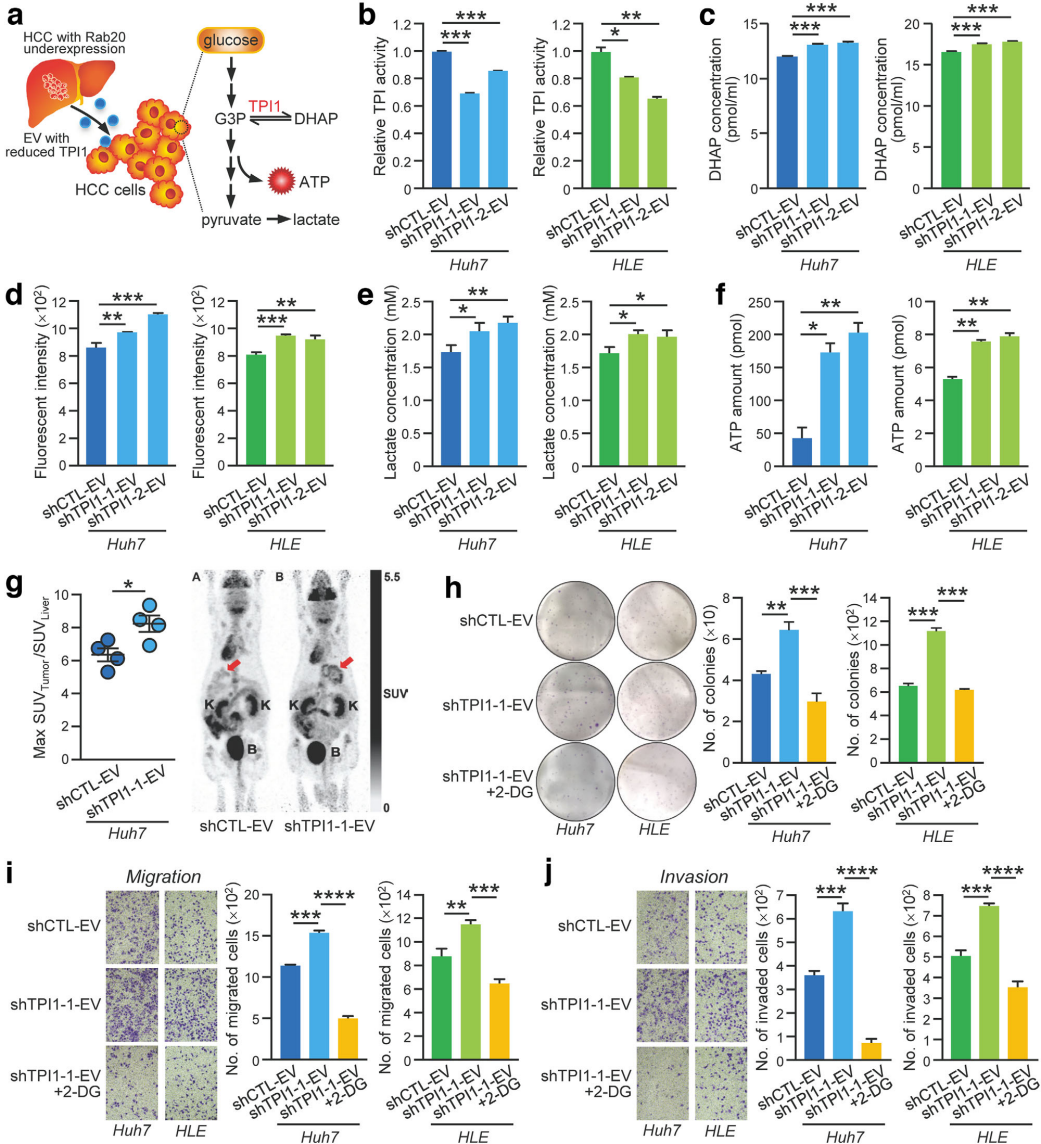
EVs with reduced TPI1 promoted aerobic glycolysis leading to enhanced growth and motility in treated cells. (a) Schematic diagram illustrating the proposed altered pathway in the recipient cells after treating with EVs with reduced TPI1. (b) After treating Huh7 and HLE cells with PLC/PRF/5 shCTL‐, shTPI1‐1 and shTPI1‐2‐EVs, TPI activity in EV‐treated cells was determined by the TPI Activity Assay. Huh7 and HLE cells treated with the indicated EVs were assayed for DHAP level (c), glucose uptake activity (d), lactate production (e) and ATP level (f). (g) Huh7 cells treated with shCTL‐ and shTPI1‐1‐EVs were orthotopically injected into the liver of nude mice (n = 4). Four weeks after the injection, ^18^F‐deoxyglucose was intravenously injected into the mice through tail vein, and the mice were subjected to micro‐PET scan to observe glucose uptake level in the liver tumour. Representative images of PET scan were displayed. The red arrow indicates the area of ^18^F‐FDG uptake in the liver. K, Kidney; B, Bladder. Huh7 and HLE cells treated with PLC/PRF/5 shCTL‐ and shTPI1‐1‐EVs in the absence and presence of 5 mM 2‐deoxyglucose (2‐DG) were subjected to colony formation (h), migration (i) and invasion (j) assays. Data are represented as mean ± SEM. *P < 0.05, **P < 0.01, ***P < 0.001, ****P < 0.0001. *P* < 0.05 is considered as statistically significant

TPI1 catalyses the interconversion of G3P and DHAP in glycolysis. We speculated that the uptake of EVs with reduced TPI1 might cause glycolytic reprogramming in the recipient cells, which in turn favours tumour progression (Figure 6a). To understand how EVs with reduced TPI1 promotes HCC, we first analysed if the activity of TPI could be detected in HLE and Huh7 cells treated with PLC/PRF/5 shCTL‐ and shTPI1‐EV. Cells treated with shCTL‐EV had lower TPI1 activity than cells treated with shTPI1‐EV (Figure 6b). Treatment of cells with shTPI1‐EV displayed higher level of DHAP when compared to cells treated with shCTL‐EV (Figure 6c). We further questioned whether cells treated with shTPI1‐EV resulted in the alteration of glucose metabolism. We found that cells treated with shTPI1‐EV showed an enhanced glucose uptake, lactate and ATP production when compared to cells treated with shCTL‐EV (Figure 6d‐6f), implying the alteration of glycolysis in the recipient cells. Effect of shTPI1‐EV on *in vivo* glucose uptake was further determined by the use of ^18^F‐FDG and micro‐PET imaging. Orthotopic liver tumours developed from shTPI1‐EV treated Huh7 cells showed a significant increase in ^18^F‐FDG uptake when compared with tumours formed from shCTL‐EV treated cells. (Figure 6g). To answer whether the enhanced aerobic glycolysis mediated by shTPI1‐EV affects HCC cell growth and motility, cells treated with shTPI1‐EV in the presence and absence of 2‐deoxy‐D‐glucose (2‐DG), a derivative of D‐glucose as a glycolytic inhibitor 2‐DG, were subjected to functional assays. The results showed that the enhancement in colony formation ability, migration and invasiveness induced by shTPI1‐EV was dampened by the cotreatment with 2‐DG (Figure 6h‐6j).

## 4 DISCUSSION

Emerging evidence suggests the potential role of membrane trafficking regulators in human cancer. As the master regulators of intracellular trafficking, Rab GTPases are widely studied for their potential functions in carcinogenesis. Re‐modelling extracellular matrix (ECM) confers a great advantage for tumour cells to metastasize to distant organs. Rab11 and Rab25 have been shown to regulate α6β4 integrin and α5β1 integrin transport, allowing hypoxia‐stimulated migration and cell migration in three‐dimensional environment, respectively (Caswell et al., 2007; Yoon et al., 2005). Through regulating protease secretion or activation, Rab7 and Rab8 are required for MT1‐MMP secretion, a tumour cell‐secreted protease that promotes ECM degradation (Bravo‐Cordero et al., 2007; Williams & Coppolino, 2011). Rab2A also controls MT1‐MMP endocytic trafficking and Golgi transport of E‐cadherin in the acquisition of a mesenchymal invasive program to promote breast cancer dissemination (Kajiho et al., 2016). Apart from the roles in tumour microenvironment modification, expression of Rabs correlates with resistance to anticancer drugs by controlling the expulsion of drugs. The presence of Rab4 and Rab6c increases anticancer drug sensitivity by reducing P‐glycoprotein level and increasing intracellular drug accumulation (Ferrándiz‐Huertas et al., 2011; Shan et al., 2000). In the physiological context, Rab3D is highly correlated with tumour malignancies in breast cancer samples. Demonstrated by functional studies, increased expression of Rab3D increases tumour invasion and distant lung metastases (Yang et al., 2015). Rab22A overexpression is detected in primary breast tumours and associated with decreased overall and metastasis‐free survival (Wang et al., 2014) whereas Rab22A knockdown impairs breast cancer metastasis in an orthotopic mouse model. Other Rab members, Rab1A, Rab5A, Rab7 and Rab27A, have been shown to be highly expressed in melanoma cells (Peinado et al., 2012).

In HCC, several Rab GTPases acquire oncogenic roles and are found to be overexpressed in tumour samples. Membrane‐bound Rab5 regulates EGF receptor endocytosis and FAK phosphorylation to control HCC cell invasiveness and growth (Fukui et al., 2007; Geng et al., 2016). By inducing the PI3K/AKT pathway, Rab31 was shown to promote tumour cell growth and reduce apoptosis (Sui et al., 2015). Rab23 is also found to be oncogenic by regulating cell growth (Liu, 2007). Conversely, Sui et al. showed that Rab17 is downregulated in liver paraneoplastic tissues and HCC samples (Qi et al., 2015). Despite the fact that Rab20 has been reported to be overexpressed in various carcinomas (Amillet et al., 2006; Habermann et al., 2011; Turner et al., 2010), little is known about the functions and mechanistic basis of Rab20 in carcinogenesis. Our current study reported a comprehensive analysis about the clinical and functional significances of Rab20 in hepatocarcinogenesis, particularly in its association with exocytosis in HCC progression. We first unveiled that Rab20 was frequently downregulated in HCC, as shown in our in‐house HCC tissues, HCC cell lines, TCGA and GEO databases. Rab20 deregulation played a pivotal role in promoting hepatocarcinogenesis. Our results showed that HCC cells with Rab20 underexpression released EVs with promoting capacity.

The linkage between Rab proteins and exocytosis has been revealed by the involvement of Rab27a and Rab27b in the regulation of EV size, transfer and docking to the recipient membrane (Ostrowski et al., 2010). Besides Rab27, Hsu and his colleagues have demonstrated that the inhibition of Rab35 may result in impaired EV secretion and accumulation of endosomal vesicle in oligodendroglial cells (Hsu et al., 2010). It is also hypothesized that Rab11 modulates EV release in cells, with the involvement of transferrin receptor (Savina et al., 2002). Particularly in cancer, an extensive analysis is conducted in understanding the association between Rab37 and exocytosis in regulating different cancer hallmarks (Cho et al., 2018; Tsai et al., 2014; Tzeng et al., 2018). Rab13 is proposed to mediate β1‐integrin exocytosis in KRAS‐dependent manner in colorectal cancer cells (Hinger et al., 2020). With respect to Rab20, a study has delineated its necessity in controlling endosome maturation and size in macrophages (Pei et al., 2015). However, its role in exocytosis remains unknown. Here, we demonstrated that the inhibitory effect induced by intracellular Rab20 expression was conferred by the release of EVs.

We proceeded to delve into the proteomic profile of EVs from Rab20 cells, which identified the preferable interaction of intracellular Rab20 and EV‐TPI1. It is intriguing to observe that the cellular TPI1 level is unaffected by Rab20 expression, yet the level of EV‐TPI1 and cellular Rab20 is positively associated. It is interesting to note that Rab20 interacts with TPI1 in cells; yet only TPI1 but not Rab20 is detected in EVs released by MIHA cells (**Figure S10**). Rab proteins plays crucial role in vesicular trafficking in cells and exosome biogenesis and secretion (Sinha et al., 2021). Rab5, an extensively studied endocytic protein, is essential for cargo packaging into clathrin‐coated pits with the assistance of clathrin and adaptor complex AP2 (Mclauchlan et al., 1998). Rab5 also recruits Class III PI3K hVps34/p150 to the early endosome (Murray et al., 2002). Rab7, the principal regulator of late endosome dynamics, interacts with VPS35 to recruit and/or stabilize VPS26:VPS29:VPS35 heterotrimer cargo selection complex on the endosome membrane and coordinate the timing of cargo export with endosome maturation (Liu et al., 2012). Furthermore, Rab9 promotes sorting of TIP47 into late endosome (Murray et al., 2005). These findings suggest the possibility of Rab20 in the regulation of cargo sorting. How Rab20 facilitates the sorting of TPI1 during the biogenesis of EVs is an interesting area to be explored.

The role of TPI1 in cancer is controversial due to its expression and functions in different cancer types (Unwin et al., 2003; Wang et al., 2008; Zhang et al., 2009). For example, TPI1 suppresses HCC cell growth and motility (Jiang et al., 2017) while high TPI1 level has been shown to be associated with gastric cancer (Chen et al., 2017). The presence and significance of TPI1 in EVs have not been reported so far. TPI1 regulates the interconversion beween DHAP and G3P in glycolysis and gluconeogenesis. We envisaged that deregulation of TPI1 causes imbalance between DHAP and G3P leading to the alteration of glucose metabolism in HCC. Indeed, our findings showed that EVs with reduced TPI1 released by cells enhanced aerobic glycolysis in the recipient HCC cells. In alignment with the *in vitro* results, suppressing TPI1 in EVs also promoted *in vivo* glucose uptake of the orthotopic liver tumours developed from EV‐treated HCC cells. Glycolysis is a metabolic pathway, that is, commonly hijacked by cancer cells to increase glucose uptake and to facilitate high rate of energy production to support the growth of cancer cells. Cancer cells favour aerobic glycolysis over mitochondrial oxidative phosphorylation even when oxygen is sufficiently provided, in order to sustain a high growth rate (Santos De Souza et al., 2011; Warburg, 1956). In HCC, increased aerobic glycolysis is frequently observed and correlated with tumour aggressiveness and poor prognosis (Kitamura et al., 2011). Using 2‐DG, an inhibitor of glycolysis, our study revealed that the HCC aggressiveness induced by TPI1‐reduced EVs was dampened.

In conclusion, our findings provide a comprehensive analysis about Rab20 downregulation in HCC and unveil an unrecognized role of Rab20 in modulating the activities of EV. We further found that the deregulation of EV‐TPI1 augments aerobic glycolysis‐driven hepatocarcinogenesis.

## FUNDING

The work was supported by the National Natural Science Fund under National Natural Science Foundation of China [Project number 82072626]; the Collaborative Research Support Scheme of State Key Laboratory of Liver Research (The University of Hong Kong) [Project number SKLLR/CRSS/2019]; and The University of Hong Kong Seed Funding for Strategic Interdisciplinary Research Scheme [Project numbers 102009863 and 007000142]. Yi Xu is supported by Hong Kong Scholars Program 2020.

## CONFLICT OF INTEREST

The authors report no conflict of interest.

## AUTHOR CONTRIBUTIONS

Bonnie Hei Man Liu performed most of the experiments, analysed data and wrote manuscript. Sze Keong Tey, Xiaowen Mao, Angel Po Yee Ma, Samuel Wan Ki Wong, Tung Him Ng, Cherlie Lot Sum Yeung, Yi Xu and Yue Yao provided technical assistance throughout the study. Eva Yi Man Fung performed mass spectrometry. Kel Vin Tan and Pek‐Lan Khong performed PET and MRI scans. Daniel Wai‐Hung Ho assisted in clinicopathological analysis. Irene Oi‐Lin Ng provided clinical samples. Alexander Hin Ning Tang performed histological analysis of tissue microarray. Shao Hang Cai and Jing Ping Yun provided tissue microarray. Bonnie Hei Man Liu and Judy Wai Ping Yam wrote and revised manuscript. Judy Wai Ping Yam coordinated and supervised the project and provided funding.

## Supporting information

Supporting information.

Supporting information.
