# Supplementary material for: TPI1‐reduced extracellular vesicles mediated by Rab20 downregulation promotes aerobic glycolysis to drive hepatocarcinogenesis
Source: J Extracell Vesicles. 2021 Aug 11;10(10):e12135. doi: 10.1002/jev2.12135 (PMC8357635; doi:10.1002/jev2.12135)
Supplement: Supplementary file 1 — Supporting information. [file JEV2-10-e12135-s001.docx]

**TPI1-reduced extracellular vesicles mediated by Rab20 downregulation promotes aerobic glycolysis to drive hepatocarcinogenesis.**

Bonnie Hei Man Liu^1^, Sze Keong Tey^1^, Xiaowen Mao^1^, Angel Po Yee Ma^1^, Cherlie Lot Sum Yeung^1^, Samuel Wan Ki Wong^1^, Tung Him Ng^1^, Yi Xu^1,2^, Yue Yao^1,3^, Eva Yi Man Fung^4^, Kel Vin Tan^5^, Pek-Lan Khong^5^, Daniel Wai-Hung Ho^1,6^, Irene Oi-Lin Ng^1,6^, Alexander Hin Ning Tang^1^, Shao Hang Cai^7^, Jing Ping Yun^8^, Judy Wai Ping Yam^1,6^

^1^Department of Pathology, Li Ka Shing Faculty of Medicine, The University of Hong Kong, Hong Kong

^2^Department of Hepatopancreatobiliary Surgery, Second Affiliated Hospital of Harbin Medical University, Harbin, China.

^3^Department of Endocrinology, Second Affiliated Hospital of Harbin Medical University, Harbin, China.

^4^Department of Chemistry, State Key Laboratory of Synthetic Chemistry, The University of Hong Kong, Hong Kong

^5^Department of Diagnostic Radiology, Queen Mary Hospital, the University of Hong Kong, Hong Kong

^6^State Key Laboratory of Liver Research (The University of Hong Kong), Hong Kong

^7^Department of Infectious Diseases, Nanfang Hospital, Southern Medical University, Guangzhou, China

^8^Department of Pathology, Sun Yat-sen University Cancer Center, Guangzhou, China

**Supplemental information**

**Supplemental Materials and Methods**

**Quantitative RT-PCR**

For each 20 μl reaction, 5 ng of complementary DNA, 1 μl predesigned TaqMan® Gene Expression assay (Applied Biosystems) for Rab20 (Hs00215134_m1), Rab3D (Hs00758197_m1), Rab30 (Hs00205577_m1) or Rab42 (Hs00900870_g1), 10 μl 2× TaqMan^TM^ Universal Master Mix (Applied Biosystems) and DEPC-treated water were mixed and added into each well on an optical 96-well plate. Human HPRT1 (Hs99999909_m1) was used as the housekeeping gene to normalize gene expression. The reaction was carried out using ABI PRISM^TM^ 7900HT system (Applied Biosystems). The following PCR condition was used: 50°C for 2 min, 95°C for 10 min, 40 cycles of 95°C for 15 s followed by 60°C for 1 min. The Ct value was determined by User Bulletin #2 ABI Prism 7900 Sequence Detection System (version 1.6). The ratio of tumorous to non-tumorous samples of HCC (T/NT) was calculated by T/NT=-2^ΔΔCt^, and displayed as copy number of Rab20 per HPRT.

**The Cancer Genome Atlas (TCGA) and Genomic Spatial Event (GSE) database analysis**

Expressions of Rab20 in fifty pairs of tumorous and corresponding non-tumorous liver tissues were extracted from the TCGA LIHC project. For the GSE database, expressions of Rab20 in tumorous and non-tumorous liver tissues were obtained from GSE14520 (platform: GPL3921) and GSE6764 via GEO2R platform (<https://www.ncbi.nlm.nih.gov/geo/geo2r/>). Values were expressed in log scale and compared across groups.

**Treatment of cells with EV for functional assays**

Prior to the treatment, HCC parental cells were seeded at approximately 50% of confluence on a 6-well plate, and allowed to settle overnight. Cells were washed with 2 ml PBS twice, and cultured in medium supplemented with 10% EV-free FBS. Ten μg of EV was added to the designated well. For treatment using 2-DG, final concentration of 5 mM 2-DG was added to the cells simulataneously with the addtion of EV. Seventy-two hr after the addtition of EV, treated cells were subjected to functional analysis.

**Soft agar assay**

Cell suspension of 1 × 10^4^ was first resuspended in 0.4% top agar mixed with DMEM-HG and FBS, then overlaid on top of 1% bottom agar mixed with DMEM-HG and FBS in 60 mm culture plate. The plate was wrapped with parafilm and cultured at 37°C for 2-3 weeks until colonies were formed. Number of colonies was counted under the microscope.

**Colony formation assay**

Cells were seeded at a density of 1 × 10^3^/well in 6-well plate and incubated at 37°C for 2 weeks, until colonies were formed. Cells were fixed, stained and counted.

**Cell migration and invasion assays**

Both assays were performed using Corning® Costar® Transwell® cell culture plate, in which a layer of Matrigel® Matrix Basement Membrane (Corning, USA) was coated on the Transwell for the invasion assay. Cells suspended in DMEM-HG medium were seeded on the upper chamber, while 10% FBS DMEM-HG medium was added in the lower chamber as chemoattractant. Number of cells seeded for these assays varied from cell line to cell line due to difference in cell phenotype. Number of cells seeded for HLE, Huh7 and PLC/PRF/5 was 1 × 10^4^, 4 × 10^4^ and 5 × 10^4^ per well, respectively. For MIHA and MHCC97L, number of cells seeded was 5 × 10^4^/well. A final concentration of 20 ng/mL human growth factor was added in the lower chamber as extra chemoattractant for MIHA cells due to the low number of migration and invaded cells. After 18 hr incubation at 37°C, migrated and invaded cells were fixed, stained and counted under the microscope.

**Fluorescent labeling of EV and uptake assay**

EVs were fluorescently labelled using PKH67 Green Fluorescent Cell Linker Kit (Sigma Aldrich) as per manufacturer’s protocol. Unbound fluorescent dyes were washed off using PBS. Labelled EVs were concentrated by ultracentrifugation at 100,000 × g for 2 hr and resuspended in PBS. To examine EV uptake by cells, HUVEC cells were treated with labelled EVs for 6 hr. After treatment, cells were fixed in 3.7% paraformaldehyde in PBS, stained with 4',6-diamidino-2-phenylindole (DAPI) (Thermo Fisher), and visualized under Carl Zeiss LSM900 laser scanning confocal microscope.

**Tube formation assay**

Wells in a 24-well plate were coated with Matrigel® growth factor reduced basement membrane matrix (Corning) on ice. Treated HUVEC suspended in non-supplemented Medium 200 were added into designated wells, while non-treated HUVEC suspended in LSGS-supplemented Medium 200 served as positive control. The assay was incubated for 6 hr at 37°C. Number of tubes formed was counted at 20× magnification under microscope.

**Sprouting assay**

HUVEC were treated with 30 μg of EVs for 72 hr in 60-mm cell culture plates before assay. EV-treated HUVEC were washed by PBS, detached from cell culture plate and resuspended in cell culture medium. To 4 ml medium containing 100,000 EV-treated HUVEC, 1 ml of methocel stock solution comprising 6 g methyl cellulose (Sigma Aldrich) in 250 ml basal medium was added. To form spheroids, 25 μl drops of the solution were incubated upside-down in a humidified cell culture incubator for 24 hr. Hanging drops were gently washed off with 10 ml PBS and collected by centrifugation at 200 × g for 5 min. The cell pellet was resuspended in 2 ml of methocel containing 20% FBS and 2 ml of collagen medium (collagen stock (Gibco) in Medium 199 (Gibco)). One ml of the spheroid-collagen mixture was added to each well of a 24-well cell culture plate and incubated at 37°C for 24 hr. The sprouting process was stopped by adding 1 ml of 4% paraformaldehyde. The angiogenic effect was assessed by measuring the cumulative length of the sprouts per spheroid using ImageJ image processing program.

**Matrigel plug assay**

PLC/PRF/5 cells of 1.5 × 10^6^ and 250 μl Growth factor-reduced Matrigel (BD Bioscience) and EV were mixed together at 4°C and injected subcutaneously into the flank of BALB/cAnN-nu mice. Mice were sacrificed 14 days post injection. Matrigel plugs were dissected and subjected to H&E staining and immunohistochemistry staining using anti-CD31 antibody (Abcam).

**TPI activity assay**

EV-treated cells were assayed for TPI activity using TPI Activity Assay Kit (Colorimetric) (ab197001, Abcam) according to manufacturer’s manual. A suspension of 1 × 10^6^ cells homogenized in ice cold TPI Assay Buffer was centrifuged at 10,000 × *g* for 5 min at 4°C. Supernatant of 2 μl was brought to a final volume of 50 μl with Assay Buffer, and added into each reaction well. Fifty μl Reaction Mix containing 44 μl Assay Buffer, 2 μl Enzyme Mix, 2 μl Developer and 2 μl Substrate were added into each reaction well. Standard curve was made by preparing reaction with increasing concentration of NADH standard (0, 2.5, 5, and 7.5 nmol/well). Absorbance at OD = 450 nm was read in a kinetic mode, every 2 min, for 20 min at 37°C by Tecan Infinite F200 microplate reader (Tecan). TPI activity of the samples was calculated according to the protocol.

**Dihydroxyacetone Phosphate (DHAP) assay**

Protocol and reagents were provided in High Sensitivity Dihydroxyacetone Phosphate Assay Kit (MAK275, Sigma-Aldrich). EV-treated cells of 1 × 10^6^ were homogenized in 100 μl of ice-cold DHAP Assay Buffer for 10 min, and centrifuged at 10,000 × *g* for 5 min. Supernatant was collected, and deproteinized with 10 kDa centrifugal filter (Amicon). For each reaction, 2 μl of deproteinized sample was brought to a final volume of 50 μl with DHAP Assay Buffer, and added into each well of a 96-well plate. Master Reaction Mix containing 43 μl DHAP Assay Buffer, 3 μl High Sensitivity Probe, 2 μl Enzyme Mix and 2 μl Developer was added into the well. Sample control was run in parallel with sample reaction by omitting the Enzyme Mix in the reaction Mix. Standard curve was prepared with adding increasing gradient (0, 2, 4, 6 and 8 μl) of 50 pmole/μl DHAP standard. The plate was incubated for 60 min at 37°C in dark, and the fluorescence intensity (λ_ex/em_ = 535/587 nm) was measured. Concentration of DHAP in samples was calculated according to the protocol.

**Glucose uptake assay**

Protocol and assay reagents were provided in EnzyFluo^TM^ Glucose Uptake Assay Kit (EFGU-100, BioAssay Systems). EV-treated cells were seeded at a density of 5 × 10^3^ per well in a 96-well plate and incubated overnight at 37°C. Cells were incubated with 5% FBS-supplemented DMEM-HG overnight, followed by 5% FBS-supplemented DMEM for 40 min to increase their glucose demand. Ten μl of 2-DG substrate was added into each well and incubated for 20 min. Cells were washed with ice cold PBS and lysed with 1% Triton X-100 NADP extraction buffer. Four standards with increasing concentration (0, 1.5, 3 and 5 μM) of 2-DG6P were prepared and added into separated wells. Fifty μl of NADPH extraction buffer and dH_2_O was added to each well with cells and standards, respectively. The plate was cooled at -20°C for 5 min and incubated at room temperature for 10 min. Ten μl of Working Reagent 1 (mixture of 10 μl Assay Buffer, 1 μl G6PDH Enzyme, 1 μl NADP) was added to all wells and the plate was incubated at 37°C for an hour. Fifty μl NADPH extraction buffer was added into all wells and the plate was incubated at 80°C for 15 min. After 50 μl NADP extraction buffer was added into all wells, the plate was cooled at -20°C for 5 min. Fifty μl of sample and standard from each well were transferred into separate wells in a black 96-well plate. Fifty μl of Working Reagent 2 (mixture of 45 μl Assay Buffer, 1 μl Enzyme A, 1 μl Enzyme B, 10 μl G6P Reagent and 5 μl Probe) was added into each well. Fluorescence intensity (λ_ex/em_ = 530/585 nm) was measured at time 0 and 20 min. Amount of NADPH was calculated based on the standard curve. A control group without any experimental procedure was included.

**ATP determination assay**

ATP level of EV-treated cells was analyzed using ATP Determination Kit (A22066, Invitrogen). In brief, 10 ml of standard reaction solution consisted of 500 μl 20× Reaction Buffer, 100 μl 0.1 M DTT, 0.5 ml 10 mM D-luciferin, 2.5 μl 5 mg/ml firefly luciferase and deionized water. Two μl of sample was mixed with 100 μl standard reaction solution and loaded into each well in 96-well plate. Standard curve was generated by adding increasing gradient (0, 1, 2 and 3 μl) of 100 nM ATP solution into reaction solution. Luminescence emitted by both experimental samples and standards were measured. Amount of ATP in the samples was calculated from the standard curve.

**L-Lactate assay**

Protocol and reagents needed were provided in EnzyChrom^TM^ L-Lactate Assay Kit (ECLC-100, BioAssay Systems). CM of 20 μl was added into each well of a 96-well plate. Standard curve was generated with adding increasing concentration of L-Lactate standard (0, 0.1, 0.2 and 0.3 mM). Working reagent was prepared by mixing 60 μl Assay Buffer, 1 μl Enzyme A, 1 μl Enzyme B, 10 μl NAD and 14 μl MTT. Working reagent for No Enzyme A sample control was prepared as above by omitting Enzyme A. Eighty μl of Working Reagent with or without Enzyme A was added into each reaction. Absorbance at OD = 565 nm of the reaction was read at time ‘zero’ and after 20-min incubation at room temperature. Amount of L-Lactate in the samples was calculated based on the standard curve and reading from No Enzyme A control.

**Western blot**

Protein lysate was quantified by Protein Assay Dye (Bio-Rad). Equal amount of denatured protein samples was fractionated on SDS-polyacrylamide gel electrophoresis (PAGE), followed by protein transfer to PVDF membrane (GE Healthcare Life). After blocking with skimmed milk, the membrane was incubated with primary antibody overnight at 4°C, followed by secondary antibody for 1 hr at room temperature, with washing with TBST in between. Antibodies against Rab20 (Abcam), TPI1 (Proteintech), β-actin (Sigma-Aldrich), Alix (Santa Cruz Biotech), TSG101 (BD Biosciences), CD9 (Abcam), GM130 (Abcam) and nucleoporin p62 (Cell Signaling Technology) were used. The membrane was incubated with Amersham ECL Select Western blotting detection reagent (GE Healthcare) and exposed onto X-ray film (Fujifilm).

**Co-immunoprecipitation**

MIHA cells were lysed with ice-cold NETN buffer (150 mM NaCl, 5 mM EDTA, 50 mM Tris, 0.2% NP-40). Protein lysate of 500 μg was incubated overnight with or without 1 μg of anti-Rab20 antibody (GeneTex). PBS-washed 50% Protein G Sepharose 4 Fast Flow beads (GE Healthcare) of 30 μl was subsequently added and incubated for 4 hr. The beads with immunoprecipitated proteins were washed 3 times with NETN prior to fractionation on SDS-PAGE. Western blotting analysis was performed using anti-Rab20 (GeneTex) and anti-TPI1 (Proteintech) antibodies.

**Immunohistochemistry (IHC) analysis**

Tissue slides of clinical specimen, xenograft and lungs were subjected to IHC staining. Following the dewaxing of slices, the specimens were incubated with 3% hydrogen-peroxide/methanol and blocked with a biotin-blocking kit (DAKO, Germany). Slides were incubated with primary antibody followed by biotinylated secondary antibody, and stained with DAKO liquid 3,3'-diaminobenzidine tetrahydrochloride. Primary antibodies used include anti-Rab20 (Abcam), anti-CD31 (Abcam) and anti-Ki67 (Dako). The slides were then counter-stained with Mayer’s hematoxylin and eosin (H&E). Stained specimens were scanned with Hamamatsu NanoZoomer S210 (Japan) for histological analysis. Intensity of staining was scored by experienced pathologists. HCC and the corresponding non-tumorous liver tissues were scored as 0, 1, 2 and 3 based on Rab20 intensity.

**Supplemental Tables**

**Table S1. Results of mass spectrometry produced by MaxQuant software.**

(Attached as separate excel file)

**Table S2. Modulated MHCC97L Rab20-EV proteins when compared to Vector-EV**

| Gene name | Protein name | Fold change | *P*-value |
| --- | --- | --- | --- |
| AHNAK | Neuroblast differentiation-associated protein AHNAK | 7.642 | 0.000004 |
| THBS3 | Thrombospondin-3 | 7.607 | 0.017477 |
| TPI1 | Triosephosphate isomerase | 6.342 | 0.040629 |
| PSMA2 | Proteasome subunit alpha type-2 | 6.293 | 0.034160 |
| CD9 | CD9 antigen | 5.732 | 0.003105 |
| RELN | Reelin | 4.697 | 0.000632 |
| B4GAT1 | Beta-1,4-glucuronyltransferase 1 | 3.586 | 0.006689 |
| MASP1 | Mannan-binding lectin serine protease 1 | 3.274 | 0.004576 |
| COL5A1 | Collagen alpha-1(V) chain | 3.197 | 0.028596 |
| CFL1 | Cofilin-1 | 3.079 | 0.002836 |
| PARVB | Beta-parvin | 2.872 | 0.045096 |
| LRP1 | Prolow-density lipoprotein receptor-related protein 1 | 2.865 | 0.000255 |
| MRC2 | C-type mannose receptor 2 | 2.837 | 0.000012 |
| KPNB1 | Importin subunit beta-1 | 2.827 | 0.003415 |
| COL1A2 | Collagen alpha-2(I) chain | 2.797 | 0.000503 |
| CNTN1 | Contactin-1 | 2.649 | 0.006134 |
| NPTX1 | Neuronal pentraxin-1 | 2.579 | 0.012273 |
| PLXDC2 | Plexin domain-containing protein 2 | 2.322 | 0.007789 |
| GDI2 | Rab GDP dissociation inhibitor beta | 2.158 | 0.004383 |
| FAT4 | Protocadherin Fat 4 | 2.157 | 0.000176 |
| LMNA | Lamin-A/C | 0.472 | 0.000137 |
| ALDH1A1 | Retinal dehydrogenase 1 | 0.451 | 0.001783 |
| TNC | Tenascin | 0.446 | 0.000185 |
| LDHA | L-lactate dehydrogenase A chain | 0.381 | 0.000743 |
| ILK | Integrin-linked protein kinase | 0.340 | 0.006306 |
| HSPB1 | Heat shock protein beta-1 | 0.332 | 0.000081 |
| ARPC2 | Actin-related protein 2/3 complex subunit 2 | 0.296 | 0.018658 |
| NACA | Nascent polypeptide-associated complex subunit alpha | 0.275 | 0.025535 |
| FGG | Fibrinogen gamma chain | 0.274 | 0.000945 |
| SPP1 | Osteopontin | 0.261 | 0.000256 |
| P4HB | Protein disulfide-isomerase | 0.243 | 0.004038 |
| PIP4K2A | Phosphatidylinositol 5-phosphate 4-kinase type-2 alpha | 0.233 | 0.000365 |
| NUTF2 | Nuclear transport factor 2 | 0.126 | 0.017535 |

**Supplemental Figure**


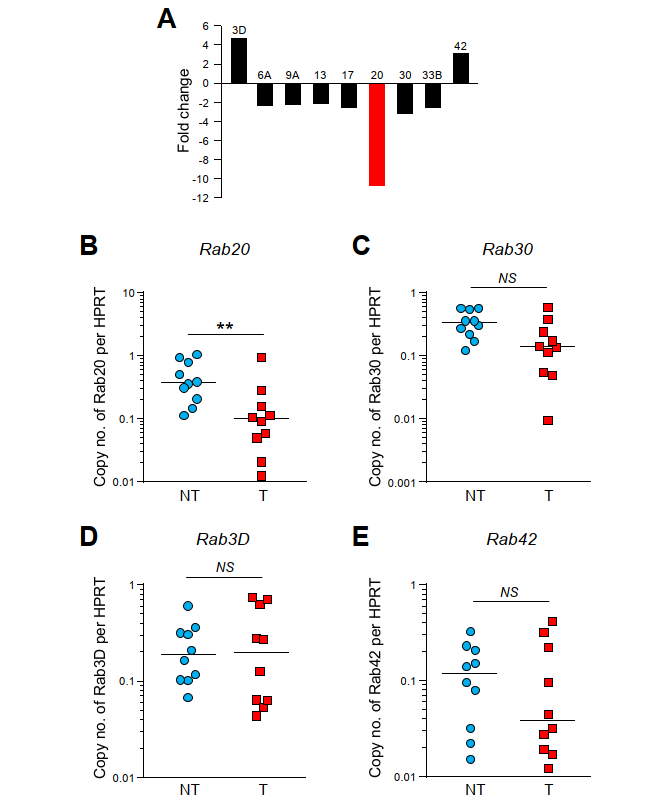


**Supplementary Figure S1. Expression of Rab GTPases in HCC.** (A) Analysis of expression of Rab GTPase family members in a pair of tumorous and adjacent non-tumorous liver samples collected from a patient with late HCC. Fold change of Rab expression was shown. (B) Quantitative RT-PCR was used to assess expressions of Rab20 (B), Rab30 (C), Rab3D (D) and Rab42 (E) in 10 pairs of HCC and non-tumorous liver tissues obtained from Queen Mary Hospital, Hong Kong. Data are represented as mean. ***P* < 0.01. *P* < 0.05 is considered as statistically significant. *NS*, not significant.


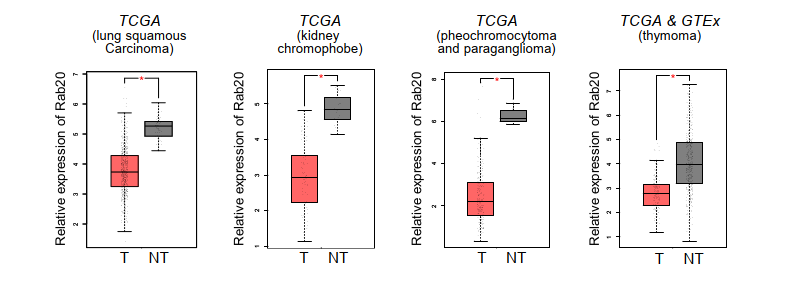


**Supplementary Figure S2. Downregulation of Rab20 in other solid tumors.** Expression levels of Rab20 in tissues with lung squamous carcinoma, kidney chromophobe, pheochromocytoma and paraganglioma, and thymoma were compared with their corresponding non-tumorous tissues using Gene Expression Profiling Interactive Analysis platform (http://gepia.cancer-pku.cn). Asterisk represents significant downregulation of Rab20 in the tumorous tissues. **P* < 0.05. *P* < 0.05 is considered as statistically significant.


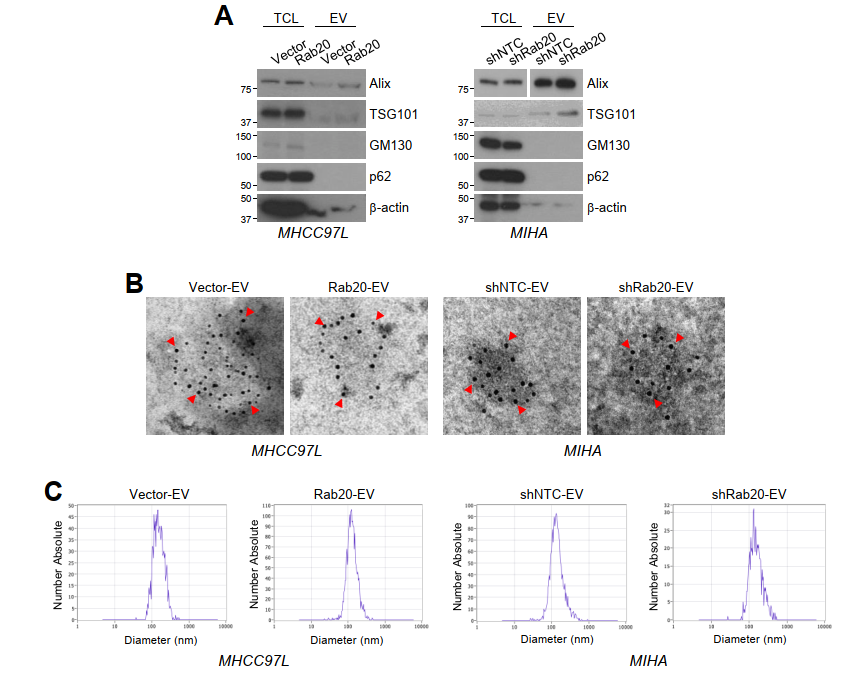


**Supplementary Figure S3. Validation of the purity and integrity of EVs of MHCC97L Vector and Rab20 cells and MIHA shNTC and shRab20 cells.** (A) Immunoblot images showing expression of EV markers in the total cell lysates (TCL) and the isolated EVs from MHCC97L Vector and Rab20 cells (*Left*), as well as MIHA shNTC and shRab20 cells (*Right*). Alix and TSG101 served as the positive markers, while GM130 and p62 were the negative markers. Loading of EV samples was normalized by the expression of β-actin. (B) EVs were deposited on Formvar-carbon coated electron-microscopy grids, and labelled with anti-CD63 antibody followed by secondary antibody coupled with gold particles. The grids were observed under electron microscope. Images of labeled EVs were captured at the magnification of 52000×. (C) EVs were subjected to size distribution measurement by the Particle Metrix Zetaview® nanoparticle tracking analyzer.


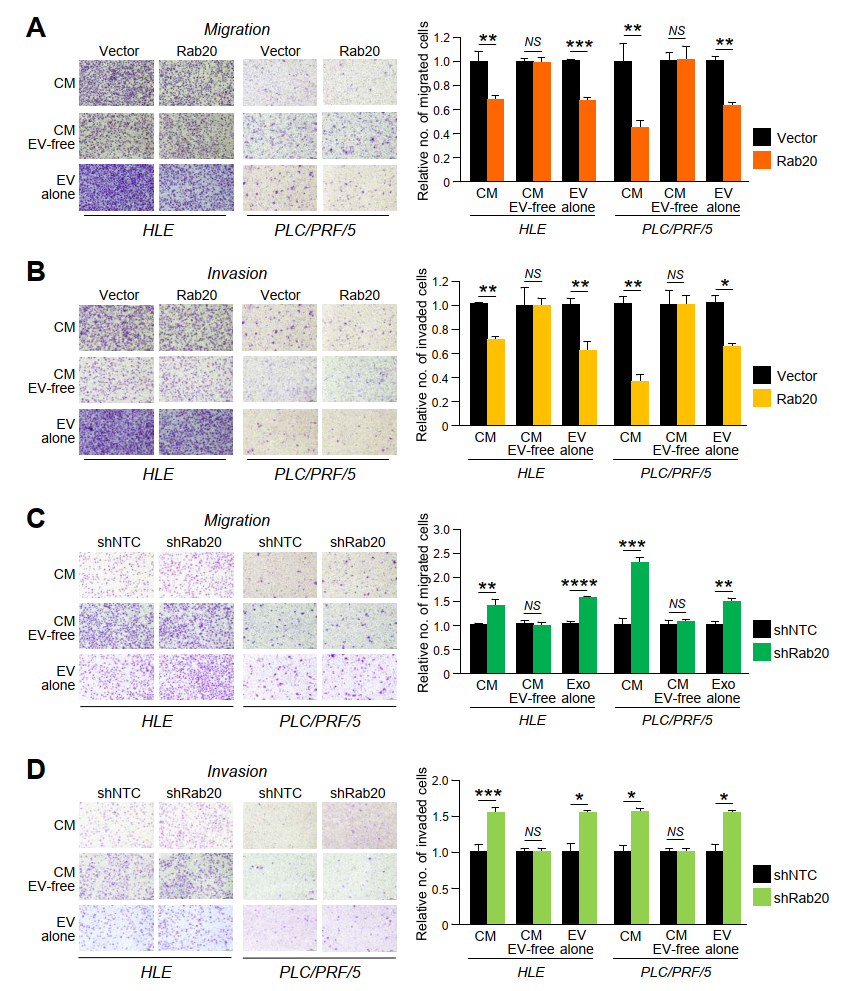


**Supplementary Figure S4. EVs are the active mediators controlled by intracellular Rab20 expression in regulating HCC.** Conditioned medium (CM) was collected by incubating MHCC97L Vector and Rab20 cells with EV-free FBS-supplemented full medium for 72 h. Using ultracentrifugation, both EVs and EV-free CM were collected. HLE and PLC/PRF/5 cells were treated with CM, EV-free CM and EVs and subjected to migration (A) and invasion (B) assays. Same approach was adopted to collect CM, EV-free CM and EVs from MIHA non-target control (shNTC) and Rab20 knockdown (shRab20) cells. HLE and PLC/PRF/5 cells treated with CM, EV-free CM and EVs were subjected to migration (C) and invasion (D) assays. Representation images of migrated and invaded cells are shown. Number of migrated and invaded cells was counted. **P* < 0.05, ***P* < 0.01, *****P* < 0.0001. *P* < 0.05 is considered as statistically significant. *NS*, not significant.


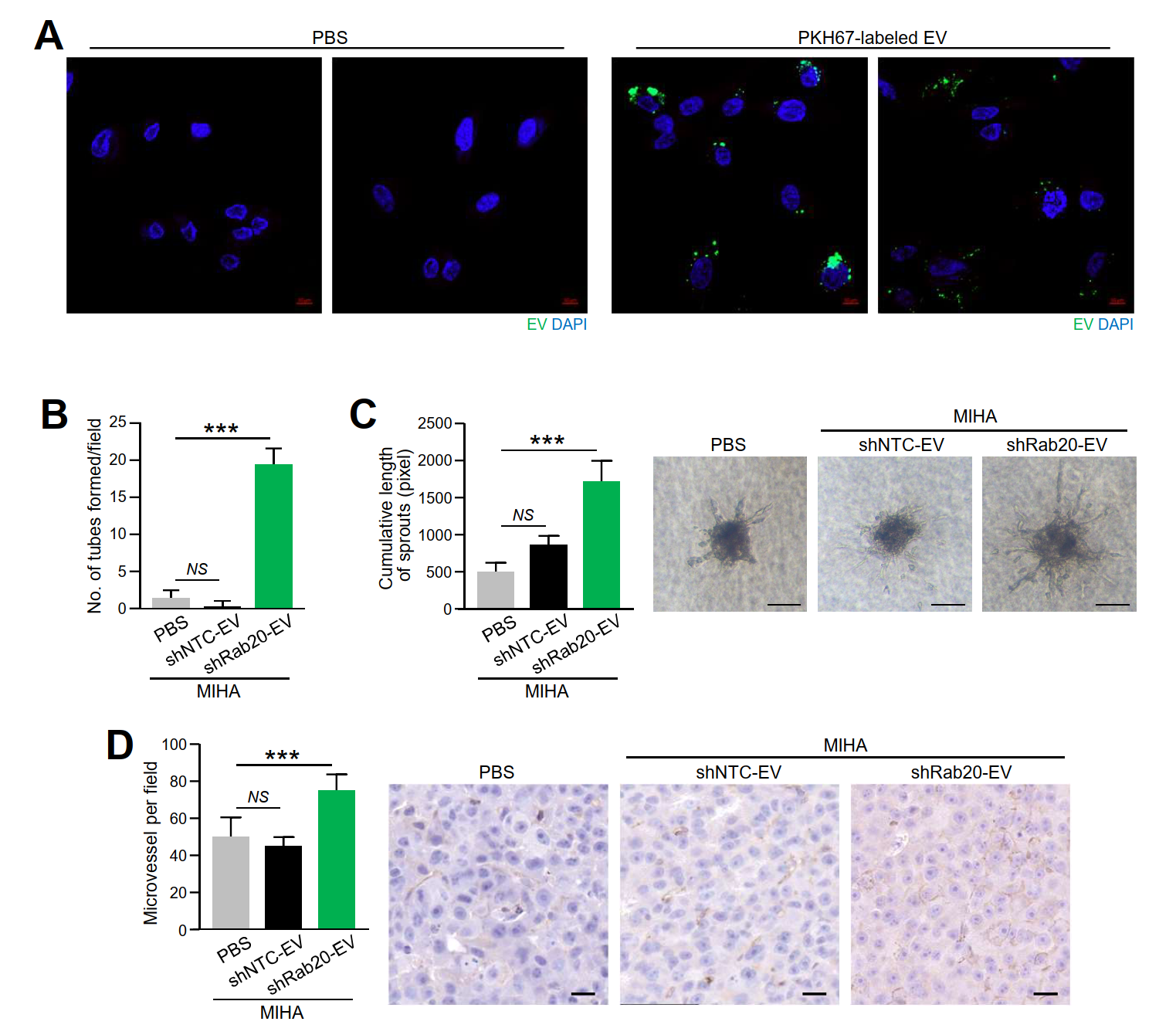


**Supplementary Figure S5. Activity of EVs in regulating angiogenesis is determined by Rab20 expression level of the releasing cells.** (A) HUVEC were incubated with or without PKH67-labeled EVs of MHCC97L cells. HUVEC was stained with DAPI and observed under fluorescent microscope. Representative images are shown. Scale bar, 10 μm. (B) HUVEC treated with MIHA shNTC- and shRab20-EV were subjected to tube formation assay. Treated HUVEC were allowed to form tubes on growth factor-reduced matrigel, and the number of tubes was counted. (C) Sprouting assay was performed using HUVEC treated with MIHA shNTC- and shRab20-EV. The newly formed sprouts were quantified. Scale bar, 100 μm. (D) Matrigel plug angiogenesis assay was carried out by coinjecting PLC/PRF/5 cells with either MIHA shNTC- or shRab20-EV. Tumors formed were dissected and subjected to immunohistochemical staining using anti-CD31 antibody. Scale bar, 50 μm.


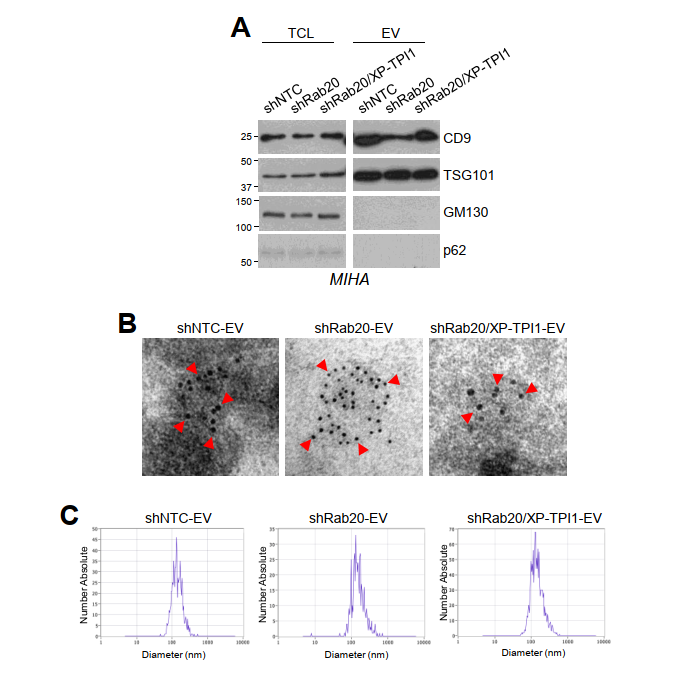


**Supplementary Figure S6. Validation of the purity and integrity of EVs isolated from MIHA shNTC, shRab20 and shRab20/XP-TPI1 cells.** (A) Total cell lysates (TCL) and isolated EVs of the indicated stable clones were immunoblotted with antibodies against EV positive markers (Alix and TSG101) and negative markers (GM130 and p62). (B) EVs were deposited on Formvar-carbon coated electron-microscopy grids, and labelled with anti-CD63 antibody followed by secondary antibody coupled with gold particles. The grids were observed under electron microscope. Images of labeled EVs were captured at the magnification of 52000×. (C) EVs were subjected to size distribution measurement by the Particle Metrix Zetaview® nanoparticle tracking analyzer.


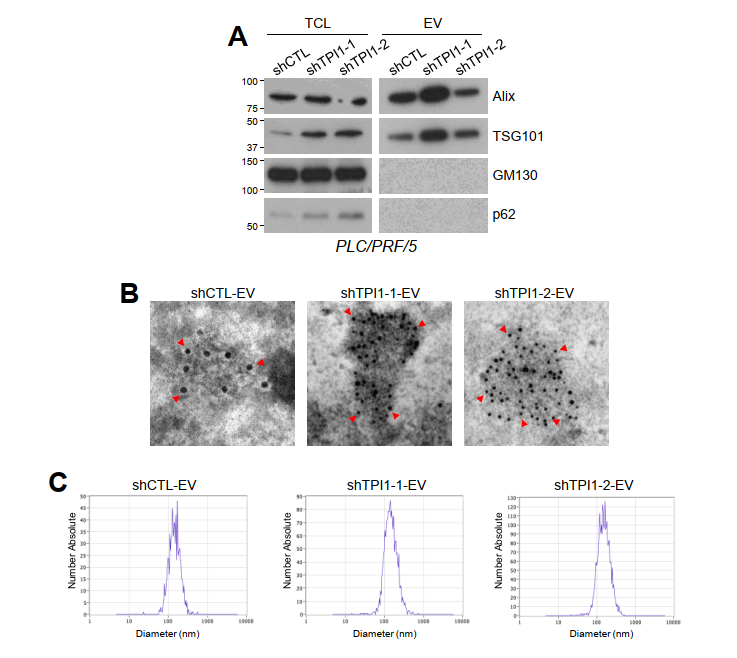


**Supplementary Figure S7. Validation of the purity and integrity of EVs isolated from PLC/PRF/5 non-target control and TPI1 knockdown cells.** (A) Immunoblot images showing expression of EV markers in the total cell lysates (TCL) and EVs collected from PLC/PRF/5 non-target (shCTL) and TPI1 knockdown (shTPI1) cells. Alix and TSG101 are the positive markers, while GM130 and p62 are the negative markers. (B) EVs were deposited on Formvar-carbon coated electron-microscopy grids, and labelled with anti-CD63 antibody followed by secondary antibody coupled with gold particles. The grids were observed under electron microscope. Images of labeled EVs were captured at the magnification of 52000×. (C) EVs were subjected to size distribution measurement by the Particle Metrix Zetaview® nanoparticle tracking analyzer.


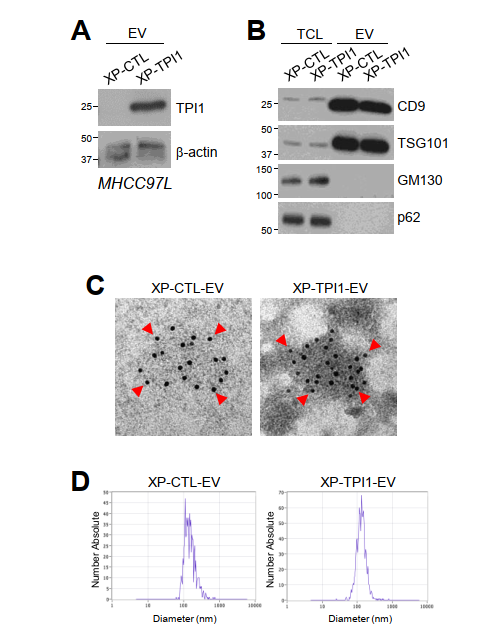


**Supplementary Figure S8. Validation of the purity and integrity of EVs isolated from MHCC97L XP control and XP-TPI1 cells.** (A) MHCC97L were stably expressed with XPack control (XP-CTL) and XPack-TPI1 (XP-TPI1) plasmids. Western blot analysis of TPI1 expression in EVs collected from the conditioned medium of stable cells. (B) Total cell lysates (TCL) and EVs were immunoblotted with antibodies against EV positive markers (Alix and TSG101) and negative markers (GM130 and p62). (C) EVs were deposited on Formvar-carbon coated electron-microscopy grids, and labelled with anti-CD63 antibody followed by secondary antibody coupled with gold particles. The grids were observed under electron microscope. Images of labeled EVs were captured at the magnification of 52000×. (D) EVs were subjected to size distribution measurement by the Particle Metrix Zetaview® nanoparticle tracking analyzer.


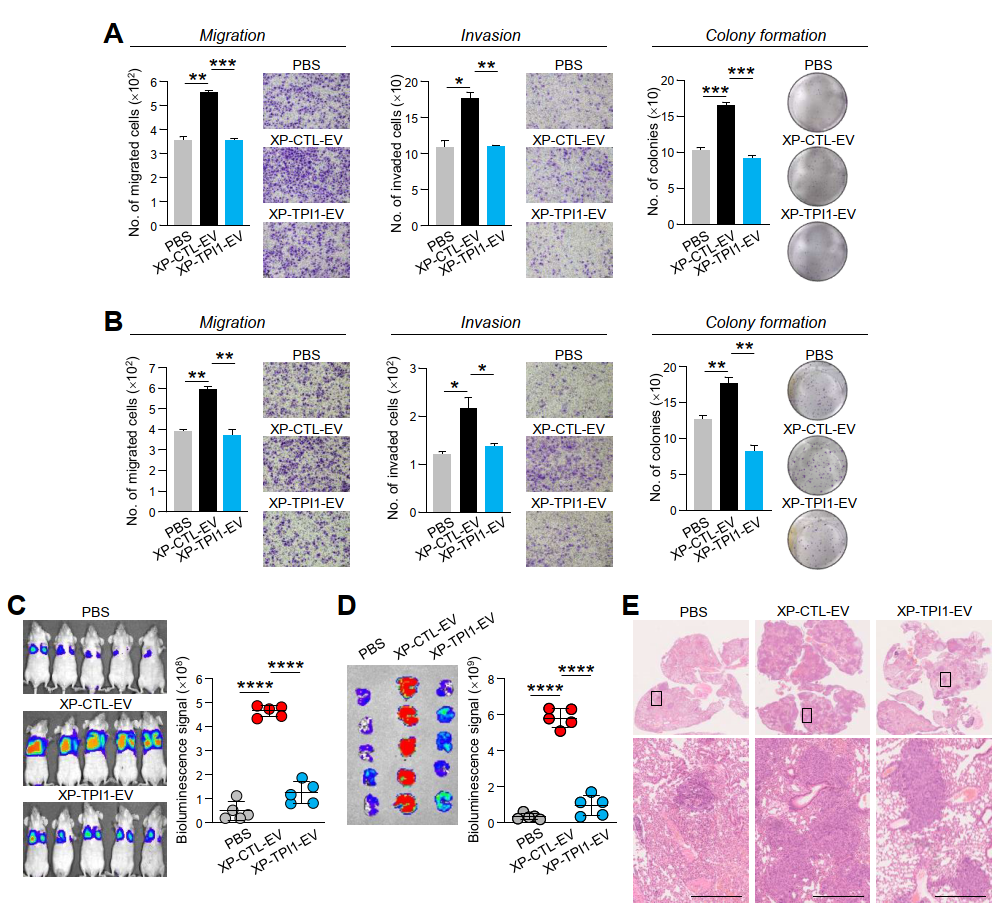


**Supplementary Figure S9. Overexpressing TPI1 in EVs increases their suppressing effect in motility, colony formation and lung colonization of cells.** Huh7 (A) and PLC/PRF/5 (B) cells treated with EVs of MHCC97L XP-CTL and XP-TPI1 cells were subjected to migration (*Left*), invasion (*Middle*) and colony formation (*Right*) assays. After incubation, cells and colonies were fixed, stained and counted. Representative images for each experimental group are shown. (C) Nude mice were intravenously coinjected with p53-/-;myc-transduced murine hepatoblasts and PBS, EVs of XP-CTL or XP-TPI1 cells. Bioluminescence imaging of animals was performed 2 weeks after injection. Intensity of signal was plotted. (D) *Ex vivo* bioluminescence imaging of the excised lungs was performed. Luciferase intensity was plotted. (E) Representative H&E-stained images showing tumor nodules in the lungs were shown. Scale bar: 500 μm. **P* < 0.05, ***P* < 0.01, ****P* < 0.001, *****P* < 0.0001. *P* < 0.05 is considered as statistically significant.


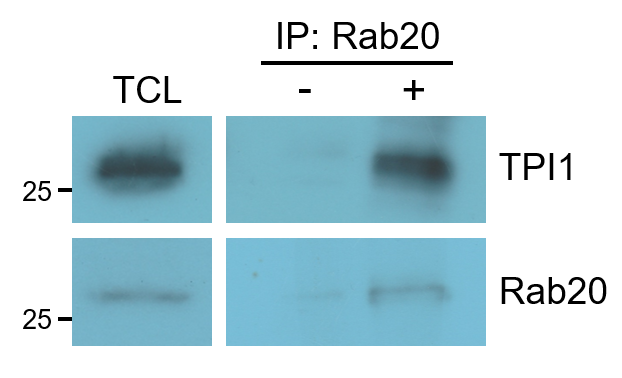


**Supplementary Figure S10. Rab20 interacts with TPI1.** Total cell lysate (TCL) of MIHA cells were immunoprecipitated (IP) with anti-Rab20 antibody and the immunoprecipitated protein lysate was subjected to immunoblotting using anti-TPI1 and anti-Rab20 antibodies.
